# Development of an Aptamer-Based qPCR Method for the Selective and Rapid Picomolar-Level Detection of Perfluorooctanesulfonic Acid in Water

**DOI:** 10.1021/acs.est.5c04730

**Published:** 2025-08-07

**Authors:** Junyoung Park, Donghyun Kim, Dongyun Kim, Jihyeun Jung, Kyung-Duk Zoh, Changha Lee, Yongju Choi, Jong Kwon Choe

**Affiliations:** 1 Department of Civil and Environmental Engineering and Institute of Construction and Environmental Engineering, 26725 Seoul National University, 1 Gwanak-ro, Gwanak-gu, Seoul 08826, Republic of Korea; 2 Department of Environmental Health Sciences, School of Public Health, Seoul National University, Seoul 08826, Republic of Korea; 3 Department of Chemical and Biological Engineering, and Institute of Chemical Process (ICP), Seoul National University, Seoul 08826, Republic of Korea

**Keywords:** aptamer-based sensing method, perfluorooctanesulfonic acid, qPCR detection, aptamer discovery, molecular dynamics simulation

## Abstract

Per- and polyfluoroalkyl substances (PFAS) are widely recognized as emerging contaminants because they are ubiquitous in various environmental media. Their potential for chronic toxicity after prolonged human exposure is a growing concern. Consequently, there is an urgent need to develop an appropriate technology to efficiently treat and rapidly and consistently monitor PFAS levels. This study reports the development of the first aptamers that can bind to perfluorooctanesulfonic acid (PFOS), with a dissociation constant (*K*_D_) of 6.76 μM, and exhibit a high specificity for PFOS even in the presence of other PFAS. The binding site and mechanism of the prepared aptamers are explored using truncation and molecular dynamics simulations, which show that the lengths of fluorocarbons and functional groups are important recognition epitopes. To demonstrate the application potential of the prepared aptamers, an aptamer-based quantitative polymerase chain reaction method is also developed, which exhibits picomolar-level detection capabilities and a limit of detection of 5.8 pM (2.9 ng/L), indicating its high sensitivity. Our findings demonstrate the potential of the developed method in the rapid *in situ* monitoring of PFOS at contamination sites, which will facilitate its early detection before rigorous analysis.

## Graphical abstract

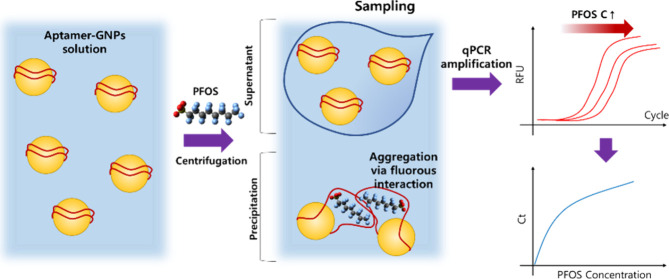

## 1 Introduction

Per- and polyfluoroalkyl substances (PFAS) are man-made organofluorine chemicals that have been widely used in industrial and household applications, e.g., as surfactants to coat products and as aqueous film-forming foams in extinguishers, since the 1950s.^1^ Their ubiquitous presence has led to the contamination of various environmental media (e.g., soil, water, and air).^2–5^ PFAS, as recognized emerging contaminants, have raised ecological concerns in the ecosystems and living organisms.^6–8^ For example, several studies have reported that the long-term bioaccumulation of PFAS can be linked to chronic toxicities in humans (e.g., thyroid disruption, immunotoxicity, low birthweight, and carcinogenic activities).^9–12^ Therefore, it is necessary to cautiously monitor PFAS levels in the environment, especially in drinking water, to maintain safe water quality. Mass spectrometry (MS)-based technologies [e.g., liquid chromatography tandem MS (LC–MS/MS) and gas chromatography–MS (GC–MS)] have been used for accurately measuring trace PFAS levels.^13^ Conventional PFAS detection methods rely on periodic sampling, which is time-consuming because of complicated pretreatment requirements, including solid-phase extraction (SPE). To continuously monitor the contamination levels of PFAS at the point-of-care, a reliable sensor or method that can monitor specific target molecules without excessive pretreatment needs to be developed.

Notably, aptamer-based sensing techniques can overcome the critical issues associated with conventional methods. Aptamers, which are uniquely coded and aligned with nucleotides, are promising biorecognition molecules that exhibit high specificity and binding affinity for target molecules. Furthermore, aptamers can be easily modified and used with various transducers (e.g., fluorescent, colorimetric, and electrochemical transducers) according to the detection needs at the point-of-care.^14–16^

In our previous study, we successfully isolated single-stranded DNA (ssDNA) aptamers and developed a fluorescence-based aptasensor for detecting perfluorooctanoic acid (PFOA), demonstrating the potential of aptamers in the detection of long-alkylated, non-hydrocarbon molecules with single functional groups.^17^ However, the developed aptamer exhibited similar binding affinities for PFOA and other PFAS compounds with fluorocarbon lengths of 6 and 7 [e.g., perfluoroheptanoic acid (PFHpA) and perfluorohexane­sulfonic acid (PFHxS)], leading to potential interference during the analysis of complex water matrices. Consequently, it is necessary to develop new aptamers with better specificity and a capability for differentiating between subtle structural variations (i.e., small differences in the fluorocarbon chain length or functional groups) among structurally similar PFAS to realize accurate quantification. Furthermore, to fully realize the practical potential of aptamers, it is essential to understand their binding mechanisms. This understanding will allow the targeted truncation of aptamer sequences, allowing the selective elimination of nonessential bases while preserving the critical structural motifs involved in binding. Optimized truncation can broaden the application prospects of aptamers across diverse detection platforms and improve their practicality by reducing their synthesis costs and complexity.^18^ For example, excessively long sequences can tightly adhere to the surface of gold nanoparticles (GNPs) used in colorimetric assays, hindering the formation of the desired aggregation-based detection signals upon target binding.^19^ Finally, because PFAS are typically in the picomolar (ng/L) concentration range in the environment,^20–22^ the developed method should be able to sensitively detect environmentally relevant PFAS levels. However, the fluorescence-based method developed in our previous study exhibited micromolar-level sensitivity, which is inadequate for detecting trace amounts of PFAS in water samples. To overcome these limitations, we hypothesized that our aptamer could be combined with a highly sensitive transduction method, e.g., quantitative polymerase chain reaction (qPCR), to considerably decrease the detection limit.^23^ Because the qPCR can amplify even picogram-level DNA, it can detect extremely low amounts of target-bound aptamers, even at the picomolar scale, enabling highly sensitive detection. To the best of our knowledge, such an aptamer-based qPCR detection strategy has been reported for only a few molecules, primarily mycotoxins (e.g., ochratoxin A,^24^ aflatoxin,^25^ and leptin^26^), and has not been used for detecting PFAS, highlighting the novelty and potential impact of our approach.

Among PFAS compounds, perfluorooctane­sulfonic acid (PFOS) raises the highest environmental concern, particularly in aquatic systems. In April 2024, the U.S. Environmental Protection Agency announced that a maximum contamination level of 4 ng/L for PFOS would be established for drinking water.^27^ Therefore, a sensitive and rapid detection method for PFOS is urgently needed to properly manage water quality. Furthermore, drinking water generally contains PFOS in the concentration range of 1–8 970 000 ng/L (geometric mean and median concentrations = 5.5 and 3.6 ng/L, respectively),^28^ depending on the water type, and PFOS often coexists with other PFAS compounds, further highlighting the need for developing a detection technique that can differentiate between PFOS and other structurally similar compounds.

Herein, we report the successful development of a DNA aptamer highly specific to PFOS and an aptamer-based qPCR detection method for the sensitive monitoring of trace PFOS levels in water. Specifically, (1) PFOS-specific aptamers are developed using the systematic evolution of ligands by exponential enrichment (SELEX) and counter-SELEX techniques by excluding DNA that can interact with structurally similar compounds (e.g., PFOA, perfluorononanoic acid (PFNA), and PFHxS). (2) The aptamer binding sites responsible for PFOS recognition are identified via aptamer truncation combined with computational analysis, and their structural influences on aptamer binding are investigated. (3) An aptamer-based qPCR detection method is developed using GNP–aptamer complexes, which are designed to enable the sensitive detection of PFOS at environmentally relevant levels (i.e., nanogram per liter). This approach is novel because it harnesses the fluorous interaction-driven precipitation^29^ of aptamer–target complexes after PFOS binding, facilitating the highly sensitive quantification of PFOS at environmentally relevant concentrations without extensive pretreatment processes (e.g., SPE).

## 2 Materials and Methods

### 2.1 Chemicals and Reagents

All chemicals and reagents used are listed in Text S1, Supporting Information.

### 2.2 Aptamer Selection and Characterization

For selection of aptamer that binds to PFOS, the SELEX method was utilized, followed by next-generation sequencing (NGS). Details on the SELEX procedure are provided in Text S2. Binding affinity of aptamers with PFOS was measured via thioflavin T (ThT) displacement method, and dissociation constant [*K*_D_] was obtained through Hill’s fitting, according to the procedure reported by Hu and Easley.^30^ Details of experimental conditions of the ThT method are provided in Text S3.

### 2.3 Molecular Dynamics Simulations

Molecular dynamics simulations (MDSs) were performed according to the procedure reported by Trinh et al.^31^ with some modifications to predict and refine the three-dimensional (3D) structures of aptamers. First, two-dimensional (2D) dot–bracket notations from Mfold^32^ were converted into RNA structures using RNAComposer.^33^ Second, they were manually modified into DNA using Discovery Studio v21 (DS). Then, the structures were further refined using Gromacs v2021.4 ^34^ with the CHARMM force field and TIP3P water model, followed by energy minimization and equilibration at 27 °C and 1 bar. The stability of the aptamers was assessed using the root-mean-square deviation (RMSD) over 10 ns, and the final aptamer structures were extracted and visualized using Visual Molecular Dynamics and DS.

AutoDock Vina^35^ was used for the molecular docking (MD) of target compounds on the aptamer to investigate their chemical interactions. MD was performed according to the method reported in our previous study.^36^ The specific conditions used for MDSs, e.g., energy minimization, temperature, and pressure equilibration, are described in Text S4.

### 2.4 Fabrication and Measurements Using the qPCR Aptasensor

GNPs were synthesized according to the modified Turkevich method^37^ with some changes. The synthesized GNPs were coated with a PFOS_JYP_6 aptamer and used for qPCR quantification. The SYBR green method was used to monitor the qPCR amplification. Details of synthesis of GNPs and aptamer-coated GNPs and qPCR conditions are described in Text S5.

### 2.5 Analytical Method

PFOS and PFAS were quantified via LC–MS/MS (Agilent 1260 Infinity II, Agilent 6470). The wastewater samples were pretreated via SPE using Oasis WAX cartridges and concentrated for accurate PFAS analysis. Additionally, inductively coupled plasma (ICP)-MS, ICP–optimal emission spectroscopy, and ion chromatography (IC) were used to analyze the cations and anions. The total organic carbon (TOC) was quantified by using a TOC analyzer. Details of LC–MS/MS conditions, ICP-MS and IC target analytes, and TOC analysis are provided in Text S6.

## 3 Results and Discussion

### 3.1 Selection of the Perfluorooctanesulfonic Acid-Binding DNA Aptamer

After nine rounds of SELEX (for a summary of each SELEX round, see Table S2), the ssDNA sequences were analyzed via NGS. Among all sequences, the seven most dominant sequences were chosen for further characterization. The PFOS-binding capabilities of these seven sequences were assessed via the ThT dye displacement method. All seven sequences exhibit higher fluorescence responses in the presence of 25 μM PFOS (Figure 1A). Furthermore, the signal changes due to PFOS addition are statistically significant across all sequences; i.e., all *p*-values are lower than 0.05 (5.58 × 10^–8^, 3.51 × 10^–8^, 1.61 × 10^–6^, 1.97 × 10^–6^, 3.6 × 10^–6^, 2.74 × 10^–8^, and 1.57 × 10^–8^ for the sequences from 1–7, respectively), according to one-way analysis of variance (ANOVA) compared to blank signals. Next, the binding affinities (using *K*_D_) were calculated using the ThT and PFOS-binding curves, as shown in Figures S1 and S2, respectively, to identify the aptamer with the best performance for subsequent investigations. As shown in Table S3, all seven sequences exhibit similar *K*_D_, ranging from 6.76 to 8.42 μM, among which PFOS_JYP_2 exhibits the lowest *K*_D_ of 6.76 ± 0.20 μM.

**Figure 1:**
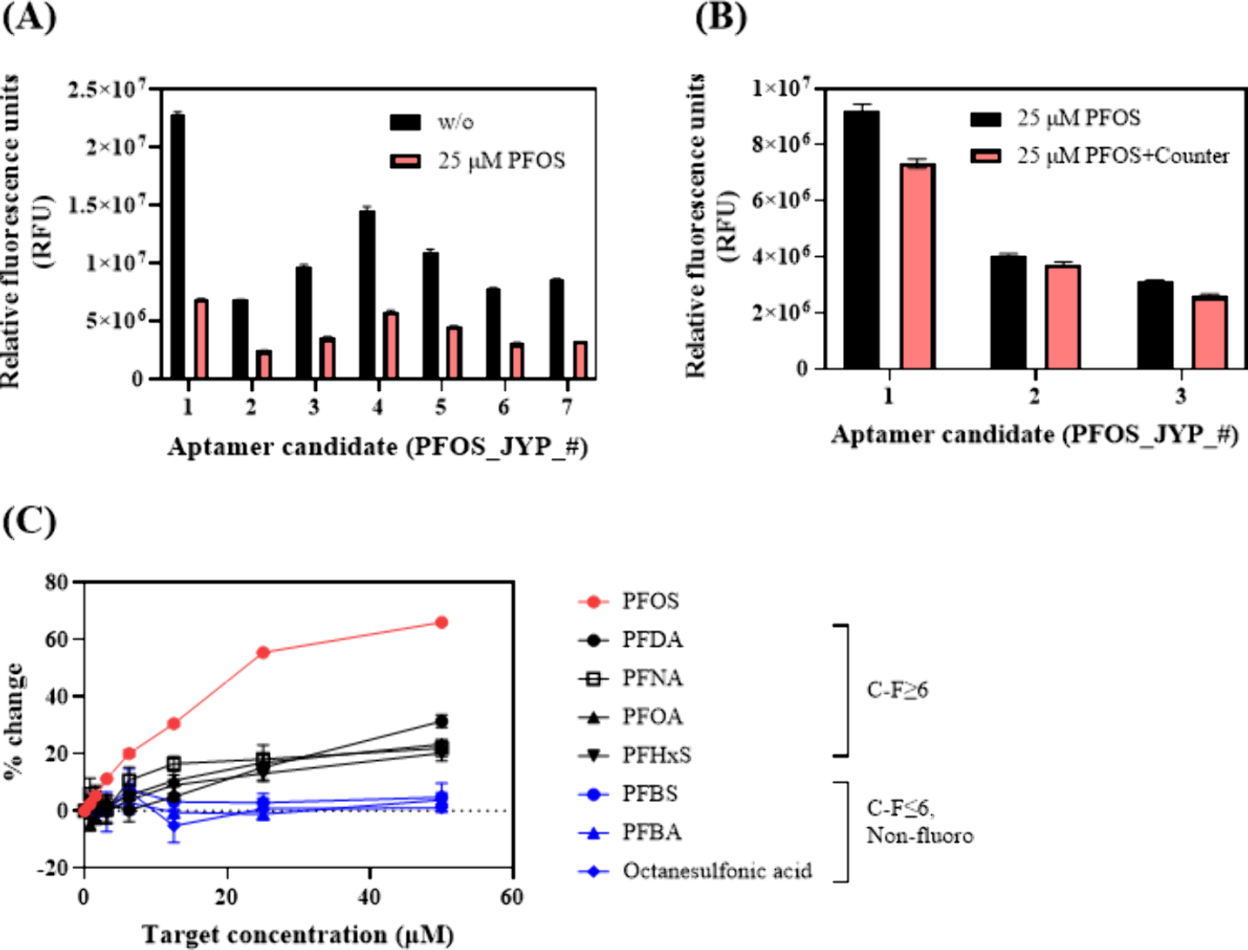
Comparison of the fluorescence responses of different aptamer candidates for (A) 25 μM PFOS and a blank solution and (B) 25 μM PFOS with and without mixed countermolecules. (C) Changes in the fluorescence responses of the PFOS_JYP_2 aptamer for a blank solution and PFOS, PFNA, PFOA, PFHxS, octanesulfonic acid, PFBS, PFBA, and PFDA with concentrations of 1.563–50 μM with 1:2 serial dilution. For all experiments, the conditions were as follows: aptamer concentration = 400 nM, ThT concentration = 10 μM, and pH = 7.5. Error bars represent the standard deviation of experimental triplicates.

Interference tests were performed by comparing the fluorescence responses of PFOS_JYP_1, PFOS_JYP_2, and PFOS_JYP_6 for 25 μM PFOS in the presence and absence of a mixture of countermolecules (i.e., PFNA, PFOA, and PFHxS; each 25 μM). According to one-way ANOVA, although only PFOS_JYP_2 exhibits a *p*-value higher than 0.05 (1.1 × 10^–2^), the fluorescence signals of PFOS_JYP_1, PFOS_JYP_2, and PFOS_JYP_6 deviate by 20.59%, 7.17%, and 17.74%, respectively (Figure 1B), indicating that structurally similar PFAS compounds do not significantly interference with aptamer–PFOS interactions under mixed conditions. Next, using PFOS_JYP_2 as the model aptamer, its cross-reactivity against different PFAS compounds with different fluorocarbon chain lengths (C–F lengths = 3–9), including octanesulfonic acid, was evaluated to gain more insights into the binding criteria (Figure 1C). Notably, PFOS_JYP_2 exhibits no measurable fluorescence response to octanesulfonic acid, perfluorobutane­sulfonic acid (PFBS), and perfluorobutanoic acid (PFBA) but exhibits a minimal response to perfluorodecanoic acid (PFDA), PFNA, PFOA, and PFHxS. These results imply that short fluorocarbon chains (<6 carbon atoms) cannot induce aptamer–target interactions. Additionally, octanesulfonic acid, a hydrocarbon analogue with functional groups and a chain length identical to PFOS, does not bind with PFOS_JYP_2, confirming that the presence of a hydrocarbon chain is not sufficient for aptamer recognition.

Furthermore, PFOS_JYP_2 exhibits similar binding capacities for long fluoroalkyl chain-containing PFAS (e.g., PFDA) and structurally similar countermolecules (e.g., PFNA, PFOA, and PFHxS), implying that a fluorocarbon chain with at least six carbons is a minimal binding requirement. The binding curves of PFOS_JYP_2 exhibit the linear detection ranges and distinct response slopes (% fluorescence change per μM) of this aptamer for PFOS (range = 0–6.25 μM, slope = 3.26), PFDA (1.56–50 μM, 0.73), PFNA (3.13–12.5 μM, 1.60), PFOA (3.13–25 μM, 0.67), and PFHxS (3.13–12.5 μM, 0.94; Figure 1C). Notably, the fluorescence response of PFOS_JYP_2 exhibits the highest increase with an increase in the PFOS concentration compared with increases in the concentrations of structurally similar PFAS compounds, indicating the superior specificity and sensitivity of PFOS_JYP_2 for PFOS. Specifically, the slopes of the fluorescence response of PFOS_JYP_2 for PFDA, PFNA, PFOA, and PFHxS are 2.0- to 4.9-fold lower than that of its fluorescence response for PFOS, indicating the higher specificity of PFOS_JYP_2 for PFOS compared to other structurally similar PFAS compounds. Additionally, the PFOS_JYP_2 aptamer showed the linear range for PFOS at a concentration starting from >0 μM, but its measurable linear response for other PFAS compounds is observed at higher concentrations (1.56–3.13 μM), further proving its specificity and relatively higher sensitivity for PFOS. Circular dichroism analysis (Figure S3) further confirms that the binding of PFOS_JYP_2 to PFOS is accompanied by distinct conformational changes, characterized by peak shifts and increased signal intensities, rather than simple adaptive binding without structural rearrangement.^38^ Next, the binding mechanism of the PFOS_JYP_2 aptamer was explored to better understand the critical factors governing aptamer–target interactions.

### 3.2 Investigation of the Binding Site and Binding Mechanism Using Truncation and MDSs

Although the aptamer specific to PFOS was identified, it was still unclear why it could bind to PFOS, i.e., the important factors affecting aptamer–PFOS binding were unclear. Before understanding the binding mechanism, the binding site on the aptamer was investigated using secondary structure and sequence homology analyses (Figure 2A). First, among the seven aptamer candidates, PFOS_JYP_1 and PFOS_JYP_2 exhibit the strongest genetic relationships, as understood from phylogenetic tree analysis. Second, homology results render 15 consensus sequences between the two aptamers, which are GGGxxxCxGTxTxxCTCxxxxGxGTxCT, where x denotes the nonconsensus bases of PFOS_JYP_2. Furthermore, PFOS_JYP_1 and PFOS_JYP_2 are structurally similar, with two loops and stems. Finally, the *K*_D_ values of the PFOS_JYP_1 and PFOS_JYP_2 aptamers are almost identical. These results indicate that high similarity in secondary structures and consensus sequences may lead to highly similar binding affinities. Therefore, we hypothesized that the upper small loop, comprising most of the consensus sequences (9 out of 15), in the aptamer would be responsible for its PFOS binding. To confirm this hypothesis, a binding test was performed for the truncated aptamer corresponding to this upper small loop using PFOS concentrations of 0–50 μM, and the *K*_D_ was calculated using ThT and PFOS-binding curves (Figure S4). The upper small loop truncated from PFOS_JYP_2 shows fluorescence responses for PFOS, with a *K*_D_ of 11.21 ± 0.16 μM (Figure 2B). Although this *K*_D_ is slightly lower than that of the original aptamer, the binding trend is highly consistent. For most concentrations, the differences in the signal changes of the two aptamers is not statistically significant (all *p*-values range from 1.56 × 10^–1^ to 2.14 × 10^–1^) except at PFOS concentrations of 3.125, 12.5, and 50 μM, where statistically significant differences are observed (*p*-values of 1.65 × 10^–4^, 2.80 × 10^–2^, and 1.78 × 10^–3^, respectively). These results confirm that the bases forming the upper small loop of PFOS_JYP_2 act as the primary binding site for PFOS. However, no fluorescence response is observed in the presence of different PFOS concentrations, particularly when the flanking region of PFOS_JYP_2 is shortened to 4 base pairs, but the sequences of the binding site are preserved (Figure S5). This lack of response is attributed to the considerable change in the secondary structure of the aptamer caused by the shortened flanking region. Briefly, the truncation results imply the importance of the folding motif and the preservation of the binding sequence for inducing aptamer–PFOS binding.

**Figure 2:**
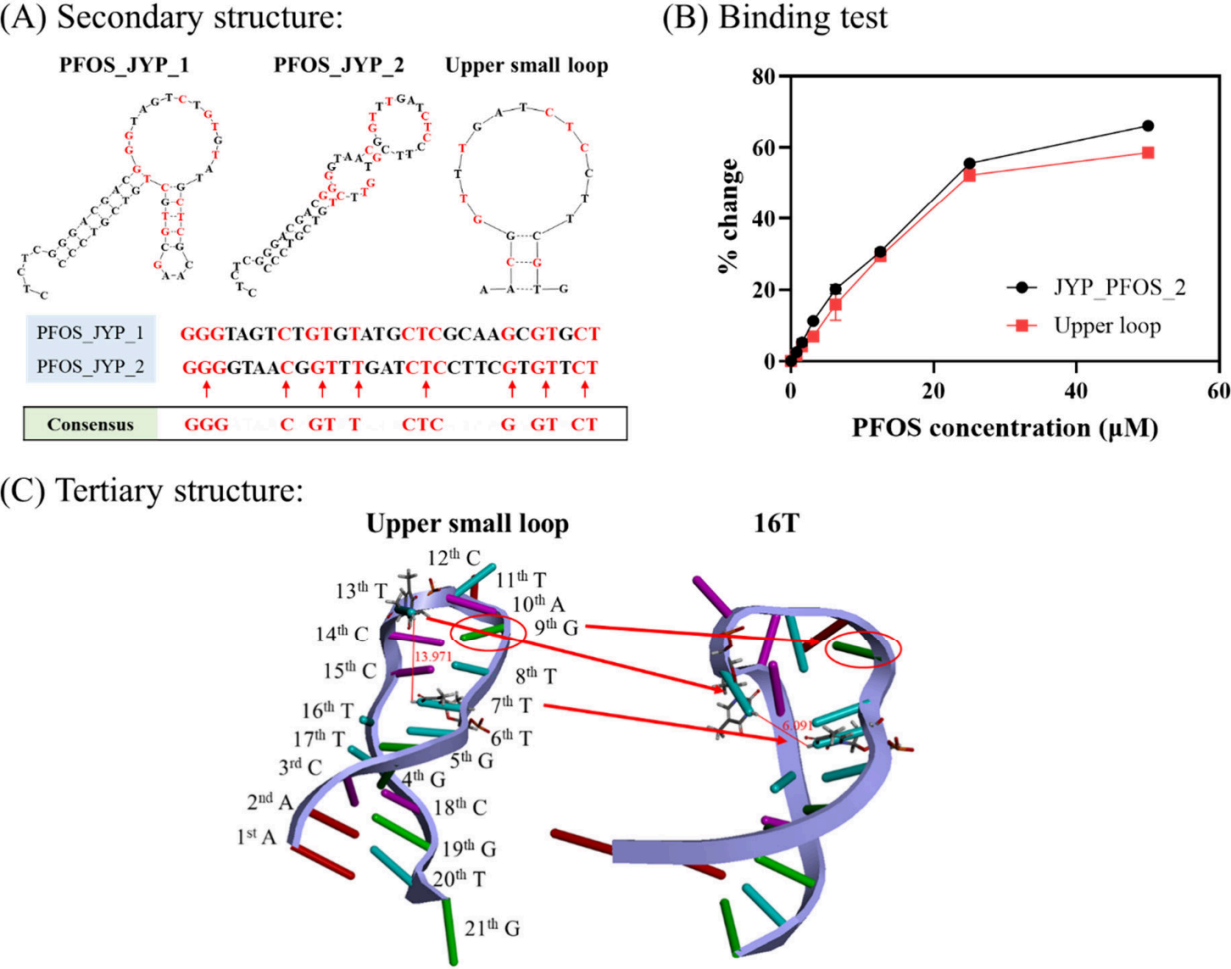
(A) Secondary structures PFOS_JYP_1 and PFOS_JYP_2 predicted using the MFold web-based program.^32^ The inset table shows the phylogenetic tree and sequence homology analyses of PFOS_JYP_1 and PFOS_JYP_2 (consensus sequences are marked in red) performed using MultAlin.^39^ (B) Changes in the fluorescence responses of PFOS_JYP_2 and the upper loop at a pH of 7.5. Herein, 0–50 μM PFOS with 1:2 serial dilution was incubated with each aptamer (400 nM) and 10 μM ThT. Error bars represent the standard deviation of experimental triplicates. (C) Predicted 3D structures of the upper small loop shown in (B) and 16T aptamers. Distances are marked in red (Å), and guanine at the ninth position is marked using a red circle.

After confirming the binding site and the critical role of the secondary structure in aptamer–PFOS interactions, we studied the origin of the observed specificity of the aptamer for PFOS over structurally similar PFAS compounds. Specifically, we aimed to understand why the aptamer exhibited minimal binding responses to certain PFAS molecules but exhibited no detectable interactions with shorter-chain or hydrocarbon-based analogues (Figure 1C). To systematically address this question, we carefully compared the chemical interactions of the aptamer with PFOS and other compounds with no (i.e., PFBS and octanesulfonic acid) or minimal (i.e., PFNA, PFOA, and PFHxS) binding responses via MD. To this end, the upper small loop (i.e., binding site) was stabilized within 2 ns with an average RMSD of 0.8 without considerable variation (Figure S6), and the 3D structure was extracted at 7 ns (Figure 2C). First, the MD of PFOS and the binding site of the aptamer exhibit various chemical interactions, e.g., a hydrogen bond between the sulfonic acid group and the primary amine of the ninth guanine at the binding site (Figure 3). Second, the MD-calculated binding energies correlate well with the observed reactivity trends. The aptamer exhibits the strongest binding energy of – 5.9 kcal/mol for PFOS, followed by weaker binding energies of −5.4, −5.5, and −5.2 kcal/mol for PFHxS, PFNA, and PFOA, respectively. By contrast, PFBS and octanesulfonic acid, which do not bind to the aptamer during experiments, exhibit notably lower binding energies of −4 and −3.8 kcal/mol, respectively. Although the differences in binding energies are modest, they agree with the experimental reactivity patterns, reinforcing the validity of the MD results. Notably, the simulation results show the importance of the fluorocarbon chain in binding specificity. The fluorocarbon chains of PFOS and other structurally similar PFAS compounds interact with the multiple sites of the aptamer, whereas the hydrocarbons in octanesulfonic acid do not form such interactions. This implies that the fluorocarbon regions serve as the primary epitopes for aptamer recognition. Additionally, MD results reveal that PFOS is the only compound that forms a π–sulfur bond with the guanine of the aptamer, a bond not observed with other PFAS compounds, including PFHxS and PFBS. The absence of this interaction with the sulfonic acid of compounds with shorter fluorocarbon chains, such as PFHxS, may be due to limited spatial alignment, which prevents guanine from interacting with the sulfonic group of PFHxS to form the π–sulfur bond. This unique bonding interaction may play an important role in the improved binding affinity and specificity of the aptamer for PFOS. Furthermore, PFOS engages more bases within the binding site of the aptamer than other PFAS compounds (except for PFOA), implying the occurrence of more comprehensive binding interactions between the aptamer and PFOS. By contrast, PFBS, which exhibits no reactivity, interacts with less than five bases of the aptamer, which may not be sufficient for the aptamer to overcome the energy barrier^40^ to induce the conformational change required to bind. That is, a fluoroalkyl chain of six or more carbon atoms may be the minimal epitope required, and the sulfonic acid functional group further facilitates binding interactions with the aptamer, explaining why the aptamer shows specificity for PFOS compared with other structurally similar PFAS compounds.

**Figure 3:**
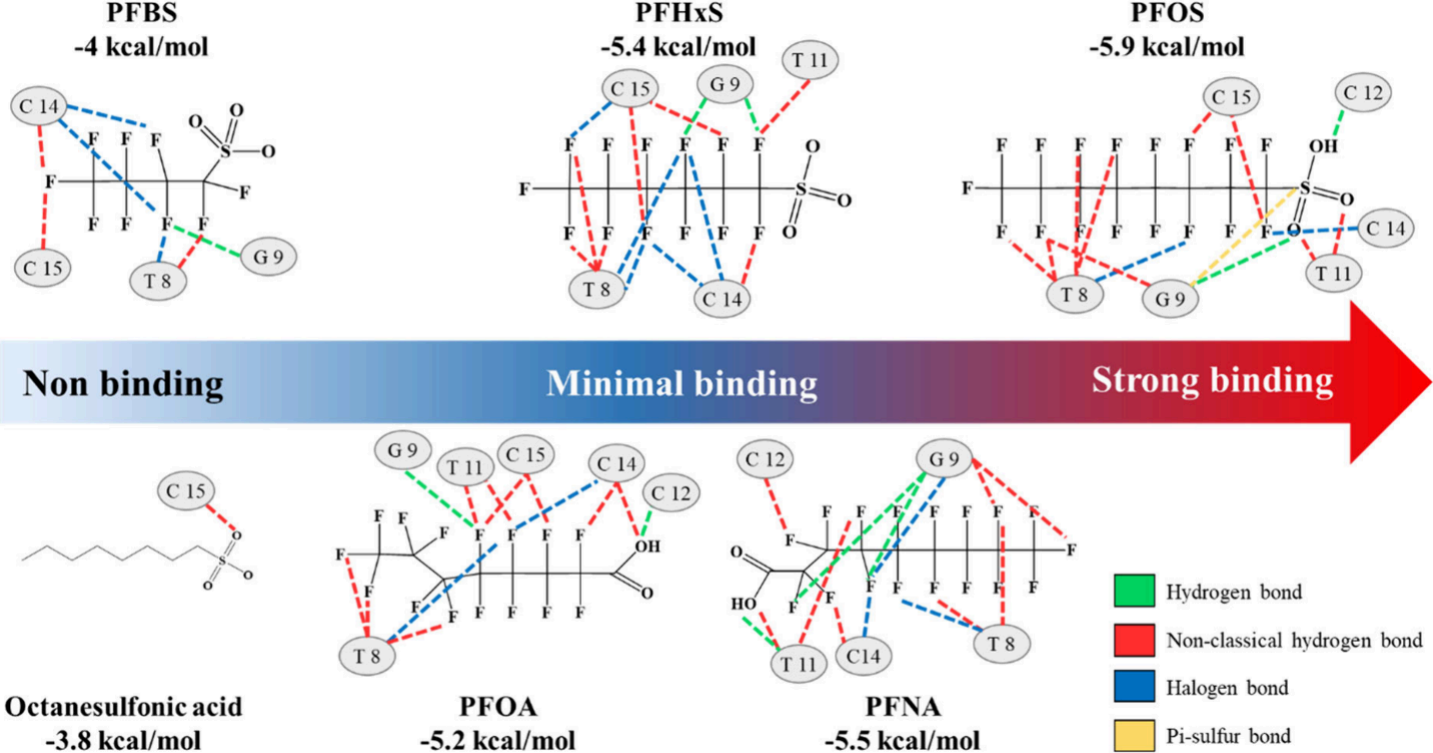
MDS results showing the binding sites of PFOS_JYP_2 for PFOS, PFNA, PFHxS, PFOA, PFBS, and octanesulfonics and the involved chemical interactions. Green, cyan, red, and yellow dashed lines represent conventional hydrogen, halogen, nonclassical hydrogen (C–H), and π–sulfur bonds, respectively.

To explore other factors influencing aptamer binding, the role of bases not identified as consensus within the investigated binding site was studied. The nonconsensus bases (highlighted in Figure S7) may engage in direct chemical interactions with PFOS but, at the same time, may still conversely influence the binding efficiency by introducing steric hindrance that prevents proper target binding.^18^ Therefore, first, a single nonconsensus base was eliminated. Then, the *K*_D_ was calculated using the ThT and PFOS-binding curves in Figures S8 and S9, respectively. All sequences with single base truncation result in improved *K*_D_ compared to the nontruncated loop shown in Table S4. This implies that the eliminated bases adversely affect the aptamer–PFOS interaction. Especially, the truncated aptamer with 16th thymine elimination (named as 16T) shows the most improved *K*_D_, almost 2-fold compared to the nontruncated loop (named the upper small loop) of PFOS_JYP_2, with values of 6.77 ± 0.16 μM and 11.21 ± 0.16 μM, respectively. To further elucidate the role of the 16th thymine in the aptamer–PFOS binding, the tertiary structures of the binding site (upper small loop) and 16T were compared via MDSs. 16T was stabilized within 3 ns with an average RMSD of 0.7 nm, without considerable variation (Figure S6), and its 3D structure was extracted at 7 ns to compare its stabilized structure with that of the upper small loop (Figure 2C). A careful study of the predicted 3D structure of the sequences shows a reduction in the loop diameter and steric hindrance. For example, the distance of the potential binding site between “TTT” (corresponds to sixth to eighth nucleotides) and “CTC” (corresponds to 12–14th nucleotides) decreases from 13.971 Å to 6.091 Å (a 2-fold decrease), specifically decreasing from hydrogen at the 3′ nitrogen of the seventh thymine to the 13th thymine. Next, the base of the 9th guanine does not block the space between the 8th thymine and the 12th cytosine/13th thymine, which are potential binding sites. This steric hindrance caused by the ninth guanine can prevent the interaction of binding sites for efficiently binding the fluorocarbon of PFOS. These results imply that the elimination of the 16th thymine decreases the distance between binding sites and reduces steric hindrance, which leads to favorable conditions for PFOS binding.

The removal of nonconsensus bases also improved the binding affinity, suggesting their presence may have exerted a more adverse effect on binding than contribution. However, the enhancement was not as significant as that observed with the 16T truncation, indicating that a contribution in binding interaction might still be necessary for optimal binding. The bases directly interacting with PFOS are illustrated in Figure S10A. Among these, the ninth guanine plays a critical role, as it directly contacts the functional group of PFOS. As shown in Table S4, the aptamer lacking the ninth guanine exhibited the smallest improvement in terms of *K*_D_ among all nonconsensus base deletions, indicating that its removal had the least impact in enhancing binding affinity. This implies that the 9th guanine is still essential for PFOS recognition. To experimentally validate this, we conducted a ThT-based fluorescence binding assay (0–50 μM PFOS, with a fixed aptamer concentration of 400 nM) using a PFOS_JYP_2 upper loop variant in which the ninth guanine was mutated to cytosine (Figure S10B,C). The resulting dissociation constant (*K*_D_) was 18.23 ± 1.92 μM, demonstrating a substantial decrease in binding affinity compared to the original sequence even when compared to the elimination of 9th guanine, referring to the more pronounced adverse effect. These results confirm the critical role of the ninth guanine in PFOS–aptamer interaction.

Based on this, we reasoned that the elimination of PFOS-interacting bases, such as the 11th thymine and 15th cytosine, would be unsuitable for truncation. Among the nonconsensus bases not directly involved in binding (i.e., the 7th thymine, 10th adenine, and 16th thymine), removal of the seventh thymine, which lies between two consensus bases (6T and 8T), alters the spacing between critical binding bases. Similarly, eliminating the 10th adenine, located between the 9th guanine and the 11th thymine (both directly involved in PFOS interaction), disrupts the local structure and may increase steric hindrance. This structural disruption is consistent and confirmed with the reduced binding affinity observed when nonconsensus bases were further removed following 16T truncation (Figures S11 and S12 and Table S5). In contrast, the 16th thymine does not intervene between consensus or PFOS-binding bases, and its removal minimally affects interbase spacing. As a result, eliminating the 16th thymine produced the lowest *K*_D_, indicating enhanced PFOS binding. Therefore, truncation at position 16 represents the most effective strategy among the single-base eliminations tested.

Briefly, these results indicate that not every base contributes positively to the aptamer–target interaction. Furthermore, the cross-reactivity test of the binding site (16T aptamer) indicates minimal responses toward PFNA, PFOA, and PFHxS, which are used in counter-SELEX (Figure S13). The similar binding trend indicates that the binding site does not lose its specificity, implying that it also regulates the specificity of the aptamer.

### 3.3 Application of the Aptamer-Based qPCR Method for Picomolar-Level PFOS Detection

An aptamer-based qPCR method was designed by immobilizing the aptamer onto the surface of GNPs via noncovalent adsorption. Theses interactions are reported to be varied depending on the ssDNA sequences^41^ and length^19^ (i.e., where the shorter ssDNA adsorb weaker) to the GNP surface. In addition, the salts should be added in accordance to screen out to the electrostatic barrier between negatively charged ssDNA and citrate-capped GNPs (150 mM Na^+^ and 2 mM MgCl_2_ were used in our study).^42^ Upon PFOS binding, only the binding site of aptamers is anticipated to undergo conformational change to bind PFOS, while nonreactive sequences remained adsorbed onto GNPs. This process results in PFOS molecules coating GNPs with the following aggregation due to the fluorous interactions. The fluorous interaction is a distinct interaction observed in perfluoroalkyl carbons, arising from the low polarizability of fluorine due to its high electronegativity and small atomic radius.^43^ As a result, perfluoroalkyl chains tend to be chemically inert toward other molecules and favor self-aggregation.^44,45^ Moreover, their characteristic helical structure promotes a more stable and tightly packed arrangement.^29,46^ These fluorous interactions drive the GNPs to cluster together, increasing the overall particle density and ultimately leading to their precipitation. That is, as the concentration of PFOS increased, fewer aptamers were available in the supernatant (representing unbound aptamers or those that did not induce sufficient aggregation) after centrifugation due to the precipitation of GNPs with high density after PFOS-binding and aggregation, requiring additional amplification cycles to achieve the fluorescence threshold (Figure 4A). Therefore, the number of amplification cycles required was proportional to the concentration of PFOS present in the solution, allowing quantitative measurement of the PFOS concentration using the cycle threshold (Ct) values obtained via qPCR. In addition, the GNPs used in this study were stabilized with citrate, initially. Next, an aptamer was presented to displace the citrate for adsorption on GNPs’ surface. No significant changes (within 12.5% deviation, Figure S14) in size were observed for 5 days after aptamer was introduced to GNPs (aptamer–GNP complexes), ensuring the high stability. This also indirectly showed that unbound sites were minimally likely to cause any disruption in terms of stability. Although the current experiment design involved the preparation of aptamer–GNP complexes before each detection, the observed stability over time represents the potential for prepacked, long-term use.

**Figure 4:**
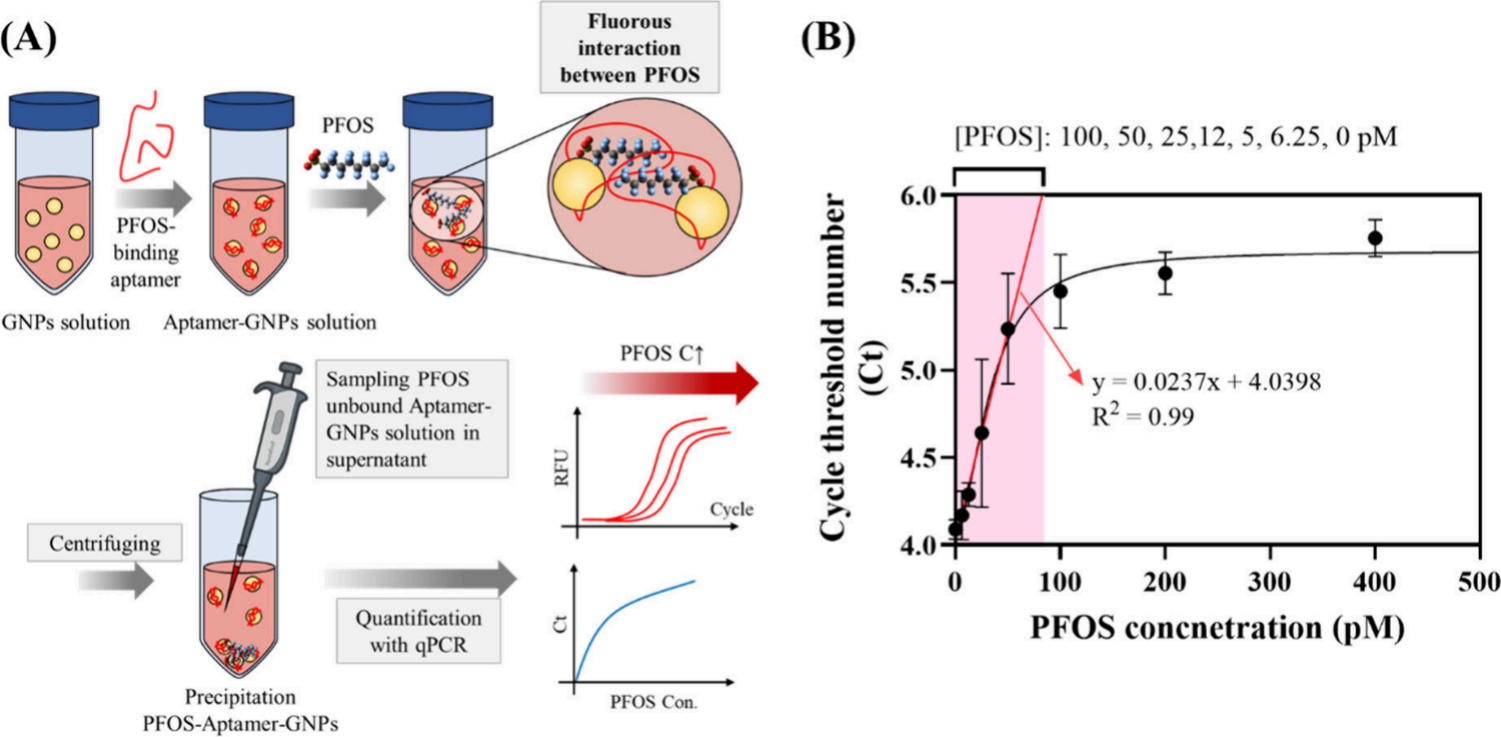
(A) Proposed design and mechanism of the qPCR-based aptasensor and (B) changes in the Ct responses in the PFOS concentration range of 0–400 pM with 1:2 serial dilution for 100 pmol of PFOS_JYP_6. The Hill’s (black line) and linear (red-dashed line) fits were used for fitting. The solution pH was maintained at 7.5. Error bars represent the standard deviation of experimental triplicates.

In the aptamer-based qPCR detection method, the selected aptamers were immobilized onto GNPs, and their responses for different PFOS concentrations (0–400 pM) were measured. Unfortunately, the two most extensively characterized aptamers (i.e., PFOS_JYP_2 and its truncated variant 16T) show no measurable responses, highlighting the necessity of exploring alternative candidates for practical sensor development. Among the other aptamer candidates, PFOS_JYP_6 exhibits a clear linear correlation between the PFOS concentration and its qPCR signal in the PFOS concentration range of 0–100 pM (*R* ^2^ = 0.99), with a low limit of detection (LOD) of 5.8 pM (2.9 ng/L), demonstrating the feasibility of using this aptamer for detecting PFOS at environmentally relevant concentrations (Figure 4B). To further validate that PFOS affects aptamer amplification in the designed qPCR method, a two-step calibration strategy was employed. First, a standard curve was established by measuring the Ct values across known concentrations of the PFOS_JYP_6 aptamer in the absence of PFOS (Figure S15A) which enabled reliable conversion of Ct values into the corresponding aptamer concentrations. Second, the amount of aptamer remaining in the supernatant after PFOS exposure (under the same concentration conditions as in Figure 4B) was quantified (Figure S15B), showing a clear concentration-dependent decrease of free aptamer as PFOS levels increased. These results further support the idea that the observed qPCR signals reflect the extent of PFOS-driven detection, allowing for sensitive and quantitative detection of PFOS via an aptamer–aggregation–qPCR mechanism. PFOS_JYP_6 may measure PFOS with high specificity because it exhibits an interference effect (17.74%) comparable with that of PFOS_JYP_2 for structurally similar PFAS compounds (Figure 1B). Despite PFOS_JYP_2 being the most extensively characterized aptamer, PFOS_JYP_6 was ultimately selected for practical PFOS detection due to its superior performance in the qPCR method. To clarify the basis for this decision, the size changes of aptamer–GNP complexes upon PFOS addition (200 pM) were measured using dynamic light scattering (DLS, Table S6). While the PFOS_JYP_6–GNP complex exhibited a 97% increase in hydrodynamic diameter, the PFOS_JYP_2–GNP complex showed a negligible change (∼9%), suggesting that PFOS_JYP_2 does not undergo a significant conformational rearrangement at its binding site upon PFOS binding. This first confirmed that JYP_PFOS_6–GNP complex could induce aggregation of GNPs in the presence of PFOS which aligned well with our proposed mechanism. To further elucidate why PFOS_JYP_6 exhibits a structural response upon PFOS interaction when adsorbed on the surface of GNPs, its potential binding site was identified using the same approach applied to PFOS_JYP_2 (Figure 2A,B) by analysis the consensus bases with PFOS_JYP_3 which exhibited almost same *K*_D_ value (Table S3). A truncated PFOS_JYP_6 containing most of the consensus sequences (8 out of 11), (small loop shown in Figure S16A) was synthesized for binding evaluation with PFOS. As a result, the percentage change in fluorescence response, measured using the ThT method (Figure S16B), increased with PFOS levels, suggesting the small loop region serves as the binding site of PFOS_JYP_6. Notably, the small loop of PFOS_JYP_6 consists of only 14 nucleotides, compared to the 21-mer loop of PFOS_JYP_2, which may facilitate easier conformational change upon PFOS binding due to the weaker adsorption on the GNPs. This structural advantage likely contributes to the higher responsiveness of PFOS_JYP_6 in the aptamer-based detection platform, ultimately making it a more suitable candidate for sensitive and reliable PFOS measurement.

Furthermore, the amplification of PFOS_JYP_6 was performed in the presence and absence of GNPs, and the melting curves are shown in Figure S17. In all cases, a single, sharp melting peak at 87.0 °C is observed, indicating that only the intended aptamer sequence is amplified, nonspecific byproducts are not formed, and primer–dimer formation does not occur. These results imply that the presence of GNPs does not interfere with the amplification to induce false signals.

Even though a consistent increase in Ct with increasing PFOS concentration is observed, the measured Ct has a high variance, causing the error bars to overlap across the samples due to the high data dispersion among the replicates, as shown in Figure S18A. This is because the absolute Ct difference across the PFOS concentration range remains relatively small (typically 0.2–0.25 cycles), with the total dynamic range of ∼1.5 cycles between blank and saturation levels. These inherently narrow signal changes may be sensitive to subtle changes such as the GNP size. Dynamic light scattering (Figure S18B) shows that the synthesized GNPs exhibit a broad size distribution with double peaks, leading to heterogeneity in aptamer immobilization and nanoparticle reactivity. Such variability in the nanoparticle size may cause differential aggregation behavior among replicates because we anticipate that the size distribution may vary slightly across the samples. However, the overall concentration dependence of the qPCR signal remains consistent across replicates, supporting the validity of the sensing mechanism.

To evaluate the practical applicability of the developed aptamer-based qPCR method in environmental monitoring, its performance was studied using a real wastewater sample collected from an industrial wastewater effluent site without spiking PFOS. The wastewater sample was heavily contaminated with various PFAS compounds, which exhibited concentrations in the range of 15 to 14 000 ng/L when measured using LC–MS/MS (Table 1). In particular, structurally similar PFAS compounds (C–F chain lengths of ≥ 6), previously identified as potential interfering factors owing to minor cross-reactivity, were detected at a total concentration of 1485 ng/L, which is >11 times higher than the PFOS concentration (130 ± 0.18 ng/L, as measured by LC–MS/MS). After pretreatment, which involved filtration through a 0.22-μm filter to remove suspended solids and a 2-fold dilution with a 2× SELEX buffer, the developed qPCR-based aptasensor is used to quantify PFOS at an average concentration of 92.4 ± 7.1 ng/L. This measurement yields an ∼29% deviation compared to the LC–MS/MS result (130 ± 0.18 ng/L shown in Table 1). While different detection platforms and specific aptamer sequences are utilized for characterization, the previously conducted specificity assessments show that structurally similar PFAS compounds, such as PFDA, induce up to ∼29% (Figure 1C) binding response relative to PFOS. Considering these previous specificity data, the observed deviation is reasonable considering the significant presence of PFAS compounds that interfere with aptamer binding to PFOS.

**Table 1:** Water Matrix of the Wastewater Sample^a^

category	compounds	concentrations quantified with LC-MS/MS
PFAS	PFBA (C–F of 3)	14000 ± 1700 ng/L
	PFBS (C–F of 4)	280 ± 1.7 ng/L
	PFHxA (C–F of 5)	920 ± 3.7 ng/L
	PFHpA (C–F of 6)	590 ± 6.8 ng/L
	PFHxS (C–F of 6)	15 ± 0.078 ng/L
	PFOA (C–F of 7)	400 ± 13 ng/L
	PFOS (C–F of 8)	130 ± 0.18 ng/L
	PFNA (C–F of 8)	290 ± 2.0 ng/L
	PFDA (C–F of 9)	190 ± 4.0 ng/L
TOC		2.2 ± 0.043 mg/L as C
pH		8.3
Conductivity (TDS)		3600 μS/cm (2300 mg/L)
Cations	Na^+^	530 ± 51 mg/L
	Si	NA
	K^+^	6.6 ± 0.53 mg/L
	Ca^2+^	170 ± 13 mg/L
	Mg^2+^	7.6 ± 0.62 mg/L
Anions	F^–^	7.6 ± 0.29 mg/L
	Cl^–^	720 ± 6.2 mg/L
	Br^–^	6.5 ± 0.26 mg/L
	NO_3_^–^	0.58 ± 0.16 mg/L
	PO_4_^3–^	NA
	SO_4_^2–^	570 ± 5.9 mg/L

a PFAS, cations, and anions were analyzed using LC-MS/MS, ICP-MS, and IC, respectively, where the specific methods were provided in Text S5. Uncertainties represent the standard deviation of experimental triplicates. Total dissolved solids (TDS) were calculated based on the conductivity multiplied by 0.64.

In addition to PFAS interference, the wastewater sample also contained extremely high concentrations of short-chained PFAS [e.g., PFBA, PFBS, and perfluorohexanoic acid (PFHxA)], with a total concentration of 15 200 ng/L, which is 117 times the PFOS concentration. Despite such extensive contamination from short-chain PFAS compounds, the observed interference (29% deviation) confirms our hypothesis regarding the minimal epitope required (C–F chain ≥ 6) for specifically binding to the aptamer. Furthermore, the wastewater sample was contaminated with substantial organic contaminants, as indicated by the measured TOC of 2.2 mg/L. Additionally, the total dissolved solid levels calculated from the measured conductivity was 2,300 mg/L, which aligns well with the measured total ion concentration (∼2020 mg/L). The robust performance of the aptamer-based qPCR method in environmental monitoring was also confirmed by spiking PFOS in two more additional real water samples, tap water and river water, as described in Text S7. Overall, the developed method successfully estimates the PFOS concentration in the picomolar range without requiring SPE pretreatment, demonstrating its potential for practical environmental monitoring applications.

### 3.4 Environmental Implications

Herein, we successfully isolated a highly specific aptamer for PFOS (*K*_D_ = 6.76 ± 0.20 μM) and elucidated key factors (i.e., sulfonic group and fluorocarbon chain length) influencing the interactions of this aptamer with target molecules (e.g., PFOS and structurally similar PFAS). The developed aptamer-based qPCR method achieved the highly sensitive detection of PFOS at picomolar concentrations (LOD = 5.8 pM or 2.9 ng/L). LC–MS/MS is considered the standard detection technique for PFOS detection because of its sensitivity (sub-ng/L) and reported accuracy (deviation within 4% with spiked levels); however, it typically requires extensive sample pretreatment resulting in long analysis times.^47^ The developed qPCR showed up to 29% deviation from LC–MS/MS analysis of real wastewater samples contaminated with multiple PFAS. This poorer accuracy compared to LC–MS/MS is mainly due to the inevitable cross-reactivity of other PFAS (total PFAS concentration is 117 times that of PFOS). However, our developed method requires no extensive pretreatment processes such as SPE, facilitating its practical use as a fast-screening tool in the early stage of monitoring for determining the approximate degree of contamination at ng/L level.

While the enzyme-linked immunosorbent assay has also been explored by others for PFOA detection, its application in PFOS detection is unclear, and the assay yields LODs of >50 mg/L, indicating its difficulty in detecting contaminants at naturally occurring levels in water.^48^ The aptamer-based qPCR method in this study exhibited higher sensitivity than other reported PFOS sensors (Table S9).^49–53^

Currently, the developed qPCR method involved a thermal cycling program of 95 °C for 5 min, followed by 45 cycles of [95 °C for 15 s, 59 °C for 30 s, and 72 °C for 45 s]. The required sample volume is only 20 μL, including 2 μL of aptamer–PFOS complexes, allowing rapid and uniform heating without a significant thermal lag. Because only up to ∼6 cycles are needed to surpass the threshold fluorescence (Ct) value for PFOS detection (Figure 4B), the analysis time can realistically be shortened to <15 min. Moreover, further optimization can considerably reduce this time, for example, decreasing the initial denaturation step from 5 min to ∼1 min according to the manufacturer’s guide of the qPCR profile, which was conservatively set to 5 min in our protocol to ensure complete denaturation. Additionally, by lowering the fluorescence threshold and reducing the number of amplification cycles required, the overall analysis time can be further decreased.

The recent advances in qPCR technology have enabled the substantial miniaturization and portability of qPCR instruments with devices now compact enough to facilitate on-site environmental monitoring. A recent study reported the development of miniaturized qPCR platforms with dimensions of only a few centimeters (i.e., size = 22 cm × 16.5 cm × 14 cm; weight ≈ 3 kg), indicating the feasibility, portability, and employability of this method in the field.^54^ Future research studies should focus on optimizing the reaction conditions and instrument design to fully recognize the rapid, portable, and high-throughput qPCR-based detection capabilities of the proposed method.

For the applications with a low power demand, our aptamer-based system can be integrated with isothermal amplification methods, such as loop-mediated isothermal amplification (LAMP)^55^ or recombinase polymerase amplification.^56^ These techniques enable nucleic acid amplification at constant temperatures (37 to 42 °C), which are widely recognized for their compatibility with field-based settings. Combining with such amplification methods is a promising solution for field-deployable PFOS monitoring platforms because of their lower power consumption. Because these methods do not require multiple temperature changes, the detection time will be decreased once the amplification duration for obtaining measurable signals is optimized.

Furthermore, additional discovery of aptamers that are specific to different PFAS compounds can be coupled with the aptamer-based qPCR platform for simultaneous monitoring of multiple PFAS targets using a single assay via the incorporation of different fluorophores in amplification using the TaqMan method because these aptamers have different sequences.^57^ Overall, this study demonstrated the aptamer-based qPCR method that can complement conventional analytical tools by offering simple, rapid, and field-deployable solutions for PFAS screening, particularly in resource-limited areas.

## Supplementary Material



## Notes

Supplementary figures (Figures S1–S19 showing spectral analysis, PFOS and aptamer binding tests over aptamer candidates, optimization conditions, tertiary and secondary structure of aptamers, truncation test, dynamic light scattering (DLS) analysis, qPCR measurement, interference tests, and mechanism validation for aptamer-based PFOS detection; Tables S1–S9 listing SELEX conditions, calculation of binding affinity (*K*_D_), qPCR condition details with melting curve and standard curves, primer sequences, molecular dynamics simulation analysis (MDS), DLS analysis, water matrix (tap and river water), and recovery data for real water samples; Texts S1–S7 on experimental protocols, instrument settings, and extended explanations including buffer composition, aptamer screening, and LC-MS/MS, IC, ICP-MS, TOC analysis (PDF)
