# Supplementary material for: Development of an Aptamer-Based qPCR Method for the Selective and Rapid Picomolar-Level Detection of Perfluorooctanesulfonic Acid in Water
Source: Environ Sci Technol. 2025 Aug 7;59(32):17247–57. doi: 10.1021/acs.est.5c04730 (PMC12368987; doi:10.1021/acs.est.5c04730)
Supplement: Supplementary file 1 [file es5c04730_si_001.pdf]

Supporting Information for

Development of an Aptamer-Based qPCR Method for the Selective and Rapid Picomolar-Level Detection of Perfluorooctanesulfonic Acid in Water

Junyoung Park[1], Donghyun Kim[1], Dongyun Kim[1], Jihyeun Jung[1], Kyung-Duk Zoh[2], Changha Lee[3], Yongju Choi[1], Jong Kwon Choe[1,*]

[1]Department of Civil and Environmental Engineering and Institute of Construction and Environmental Engineering, Seoul National University, 1 Gwanak-ro Gwanak-gu Seoul 08826, Republic of Korea

[2]Department of Environmental Health Sciences, School of Public Health, Seoul National University, Seoul, 08826, Republic of Korea

[3]Department of Chemical and Biological Engineering, and Institute of Chemical Process (ICP), Seoul National University, Seoul, 08826, Republic of Korea

**Corresponding author*: Tel.:+82 2 880 2278; Fax: +82 2 873 2684; E-mail address: jkchoe@snu.ac.kr (J. Choe)

Postal address: 35-402, Seoul National University, 1 Gwanak-ro, Gwanak-gu, Seoul 08826, Republic of Korea

Summary: 25 pages, 7 texts, 19 figures, 9 tables.

Text S1. Chemicals and Reagents. Heptadecafluorooctanesulfonic acid potassium salt (PFOS, 2795-39-3), tridecafluorohexane-1-sulfonic acid potassium salt (PFHxS, 3871-99-6), perfluorooctanoic acid (PFOA, 335-67-1), perfluorodecanoic aicd (PFDA, 335-76-2), nonafluorobutane-1-sulfonic acid (PFBS, 375-73-5), 1-octanesulfonic acid sodium salt(5324-84-5), 2M magnesium chloride solution ($MgCl_2$, 7786-30-3), and thioflavin T (ThT, 2390-54-7), and absolute ethanol(64-17-5) were purchased from Sigma-Aldrich, Inc., USA. Perfluorononanoic acid (PFNA, 375-95-1), 10X PBS solution (80.6 mM Na_3PO_4, 19.4 mM KH_2PO4, 27 mM KCl, 1.37 M NaCl, and pH 7.4), sodium hydroxide(1310-73-2), streptavidin coated agarose beads (Pierce™ Streptavidin Agarose), and 1N sulfuric acid solution(7664-93-9) were purchased from Thermo Fischer Scientific, Inc., USA. Hydrochloric acid (7647-01-0, 35.0-37.0%) was purchased from Daejung Chemicals & Metals, South Korea. Polymerase chain reaction (PCR) kit (Taq polymerase, dNTPs mixture, $MgCl_2$, and 10X PCR buffer) was purchased from Promega Corp., USA. native PFASs standard solution (2000 ng/mL), and 13C-labeled PFASs internal standard solution (2000 ng/mL) were purchased from Wellington Laboratories, Guelph, ON, Canada.

All the oligonucleotides were purchased from either GenoTech Copr. or Bioneer Cop., South Korea. Minigravity column (Micro Bio-Spin™ Chromatography Columns) was purchased from Bio-Read Laboratories, Inc., USA. Centrifugal 3 kDa MWCO and 10 kDa MWCO were purchased from Millipore, Inc., USA.

All the aqueous solutions were prepared using distilled water (UltraPure™ DNase/RNase-Free Distilled Water) purchased from Thermo Fischer Scientific, Inc., USA. Water(7732-18-5), methanol(67-56-1), and ammonium acetate (LC-MS grade) were used for LC-MS/MS analysis purchased from Sigma-Aldrich, Inc., USA.

Text S2. Discovery of PFOS Binding Aptamer. Aptamer selection was conducted via systematic evolution of ligands by exponential enrichment (SELEX) following the method of Yang et al.[1] with some modifications. This method was successfully used in our previous study to isolate PFOA binding aptamer.[2] The sequences used for SELEX is described in Table S1. SELEX is categorized into 4 steps which are isolation, amplification, strand separation, and sequencing.

From the DNA pools of each rounds, 100 pmole of random sequenced single stranded DNA (ssDNA) and 500 pmole of capture DNA (cDNA) in 250 µL of SELEX buffer (1X PBS buffer with 2 mM $MgCl_2$) were undergone for denaturation at 95°C for 6 minutes. Then, ssDNA was hybridized with cDNA at the room temperature (RT) for 30 minutes. The DNA complex was then immobilized onto the 250 µL of streptavidin agarose beads in the minigravity column. During each SELEX round, the immobilized column was washed with SELEX buffer 10 times with gradual increase up to 20 times for the subsequent rounds to remove unbound or weakly bound ssDNA sequences with higher stringency[1]. Unlike previous protocols that did not employ-Counter SELEX, a mixture of counter molecules (PFNA, PFOA, and PFHxS) was applied to the column, followed by an additional 10 washes with buffer to remove any non-specific residuals. Finally, ssDNA sequences were eluted by applying PFOS solution (PFOS in SELEX buffer). The specific levels of mixture of counter molecules and PFOS across the SELEX rounds were summarized in Table S2.

2.5 µL of each elution from final washing, counter molecules, and PFOS was then amplified via polymerase chain reaction (PCR) using thermal cycler (Tone 96, Biometra, Germany) with following temperature profile: 95°C for 2 minutes→ [92°C for 15 seconds; 59°C

for 30 seconds; 72℃ for 45 seconds]$_N$ → 72℃ for 2 minutes; 4℃. N refers to the number PCR cycles used in this study (9-13 cycles), and the PCR solutions were prepared with provided manufacturer's guide. After amplification steps, all the PCR products were put into through 3% agarose gel electrophoresis performed for 20 minutes with 100V. Stained with ethidium bromide (EtBr), the bands of each product were then finally observed under the gel documentation system. From the images, iteration of SELEX rounds was performed until the satisfied gel profile was obtained; it was repeated until the band intensity of ssDNA eluted by counter molecules no longer appears to ensure that the pools do not consist any ssDNA that binds to structurally similar molecules. At the same time, the band intensities of ssDNA eluted by PFOS solution should be stronger than these intensities of final washes solutions, which indirectly indicate that the sufficient amount of PFOS binding ssDNA were discovered.

Following the gel electrophoresis analysis, the remaining PFOS-eluted solution was concentrated using a 3 kDa molecular cut-off (MWCO) filter along with additional washes with distilled water to remove any residual PFOS. The same PCR conditions used for gel analysis were applied, except that a biotinylated reverse primer (Table S1) was used to amplify the PFOS-bound ssDNA. To prepare the ssDNA for the subsequent round, strand separation was performed to isolate ssDNA from the double-stranded PCR products. Briefly, PCR product of PFOS eluted solutions were concentrated with centrifugal 10 kDa MWCO with 200 μL of 1X PBS buffer exchange. The concentrated products were then applied into the minigravity column filled with 200 μL of streptavidin agarose beads. 10 times of washing with 200 μL of 1X PBS were applied to remove any residual PCR components (e.g., primers, dNTPs, and Taq).

Finally, 0.25 M of NaOH solutions were incubated within the column for 10 minutes to induce the DNA separation. Collected ssDNA were adjusted with 0.2 M HCl in the 2X SELEX buffer solution to reach pH 7.5. The solution was concentrated with centrifugal 3 kDa MWCO, and the pool was prepared for the next round of SELEX. The concentration of discovered ssDNA was measured using nanophotometer (P300, Implen, Germany).

The sequences of ssDNA were analyzed via next generation sequencing (NGS) in Macrogen, South Korea. NGS is a novel sequencing technology to analyze DNA or RNA sequences; in this study, the analysis was specifically performed using Illumina sequencing, identifying emitted fluorescence via fluorophore modified bases in the template DNA to analyze DNA sequences simultaneously. Through NGS, the entire sequences in the ssDNA pool were identified with corresponding frequency counts.

Text S3. ThT Method. For the calculation of binding affinity (dissociation constant, K_D) and PFOS measurement for characterization, the thioflavin T (ThT) displacement method was used. ThT is well-known dye molecule which emits the fluorescence at ~480 nm with excitation at ~430 nm once the torsional rotation is suppressed by DNA.[3] The dye could bind to cavities of folded DNA such as G-quadruplex, guanine-rich, internal gaps resulted from base mismatch, and i-motifs.[3,4] The method is a very simple tool enabling fast screening aptamer characteristic (e.g., binding affinity) with low costs. In brief, the target molecules induce aptamer to release ThT into the solvent by replacement; thus, the fluorescence decreases as levels of target molecules increase (Scheme S.1). The fluorescence was read in the emission of 480 nm at the excitation of 420 nm. Two curves from the ThT binding and target binding test with selected aptamers were plotted with Hill's fitting. For ThT binding test, fluorescence responses at 0-50 μM ThT solutions at fixed 400 nM of aptamer were recorded. For target binding test, fluorescence responses at 0 – 50 μM target solutions at fixed 400 nM aptamer and 10 μM ThT were recorded. Both tests were measured after 70 minutes of incubation at RT. Based on the binding plots, the dissociation constant (K_D) was calculated following Hu and Easley[5], and the details were described in our previous study[2].

Text S4. Molecular Dynamics Simulations. The procedure was conducted following the method described in Trinh et al.[6] with some modifications. First, 2D dot-bracket notations of aptamers from "Mfold"[7] were converted into RNA for 3D structure prediction via "RNAComposer."[8] Next, the RNA was then converted into DNA by manually changing sugar moiety (from ribose to deoxyribose) with following conversion of uracil into thymine (addition of methyl group onto the C5 of uracil) using discovery studio v21 (DS). Finally, 3D DNA structures were refined via "Gromacs v2021.4"[9] with a force-field of Chemistry at HARvard Macromolecular Mechanics (CHARMM)27. The transferable intermolecular potential three-point (TIP3P) water model with dodecahedron was applied for the solvation following with addition of counter ions (Na^+) for neutralization. After the energy minimization using steepest-descent algorithm, the equilibration was conducted at the temperature of 27℃ for constant number of particles, volume, and temperature (NVT) and at the pressure of 1 bar for particles, pressure, and temperature (NPT). Both NVT and NPT were conducted for 1 ns. After the equilibration, the aptamers were finally ran through molecular dynamics (MD) simulation. The stability was analyzed via root mean square deviation (RMSD) over 10 ns. Then the simulated aptamers were extracted using "visual molecular dynamics (VMD)"[10] and visualized in DS.

Text S5. Fabrication and Measurements Using the qPCR Aptasensor. GNPs were synthesized according to the modified Turkevich method[37] with some changes. A 20-mL aqueous solution containing 1.5 mM $HAuCl_4$ was heated in a 40-mL glass vial on a hotplate stirrer using a heating mantle under vigorous stirring. Then, 300 μL of a 0.1 M sodium citrate solution was quickly added to the boiling $HAuCl_4$ solution. The mixture was continuously boiled and stirred for 15 min to facilitate gold (Au) reduction and GNP formation.

The synthesized GNPs (total volume = 100 μL) were washed twice and centrifuged at 13,000 rpm for 5 min using 1× phosphate-buffered saline (PBS; containing 2 mM $MgCl_2$) to remove excess citrate and maintain the solution pH at 7.5. After resuspending GNPs in 1× PBS *via* vortexing and ultrasonication, a PFOS_JYP_6 aptamer (100 pmol; sequence = 5′-GGAGGCTCTCGGGACGACGGCAGCTCAGCATCTTTGTTGCCCCTGACTGTCGTCC CGATGCTGCAATCGTAA-3′) was added and incubated for 30 min. Next, the excess aptamer in the supernatant was discarded *via* centrifugation at 13,000 rpm for 5 min. Then, the resuspended aptamer–GNP complex was incubated with 200 μL of the samples containing different PFOS concentrations for 15 min. The supernatant was collected after centrifugation at 13,000 rpm for 13 min and used for the qPCR. The qPCR kit (AccuPower 2X GreenStar qPCR Master Mix, Bioneer, Korea) was prepared according to the manufacturer's guide, and amplification was performed using the following temperature profile: 95°C for 5 min → [95°C for 15 s, 59°C for 30 s, and 72°C for 45 s]$_{45\ \text{cycles}}$. The SYBR green method was used to monitor qPCR amplification; at the same time, the melting curve of aptamer in the temperature range of 65°C–95°C was analyzed to assess the specificity of the amplification.

Text S6. Analytical Method. The sizes of gold nanoparticles (GNPs) after synthesis, aptamer-conjugation, and sample incubation were measured using dynamic light scattering (DLS, Zetasizer Nano ZS, Malvern Panalytical) at the room temperature (RT) along with measurement angle of 90°. The parameters were set as follows: GNPs' refractive index (RI) of 0.2, GNP's absorption value of 3.32, the solvent viscosity of 0.8872 mPa·s, and the solvent RI of 1.33.

The LC system (1260 Infinity II, Agilent, USA) with mass spectrometry (Agilent 6120DW, Agilent, USA) was used to analyzed PFOS (m/z 499) spiked tap water samples via negative mode of electrospray ionization MS. Following gradient profile was used for measurement via ZORBAX SB C18 column (2.1 × 50 mm, 1.8 μm), and acetonitrile (eluent A) and 2 mM ammonium acetate in water (eluent B) as the mobile phase eluents: starting with 30% A (acetonitrile) at t = 0 min, increasing to 65% A for 3 min, holding at 65% A for 1 min, increasing to 100% A from 4 min to 7 min, and re-equilibrating from 8 min to 18 min. For LC conditions, 5 μL of sample was injected under the column temperature of 30 °C at 0.2 mL/min of elution flow rate. Drying gas of N_2 was supplied at the flow rate of 7.0 L/min along with drying gas temperature of 350 ºC; nebulizer pressure of 50 psi, capillary voltage of 1500 V and 4500 V for positive and negative, respectively, and fragmentor voltages of 80 V were set as MS conditions.

For LC-MS/MS analysis and aptamer-based qPCR analysis, all samples were filtered with 0.22 μm PES syringe filter before measurment. The LC system (1260 infinity II, Agilent, UA) with triple quadrupole mass spectrometry (Agilent 6470, Agilent, UA) was used to analyze PFASs in wastewater via multiple reaction monitoring (MRM) mode. PFAS was separated by ZORBAX RRHT StableBond C18 column (2.1 × 50 mm, 1.9 μm) with

InfinityLab PFC Delay Column (4.6 × 30 mm). Following gradient was used for measurement via methanol (eluent A) and 20 mM ammonium acetate in water (eluent B) as the mobile phase eluents: starting with 10% A at t = 0 min, holding at 10% A for 0.1 min, increasing to 30% A from 0.1 min to 1 min, increasing to 95% A for 3 min, increasing to 100% A for 0.5 min, holding at 100% A from 4.5 min to 6 min, decreasing to 10% A for 0.1 min, and holding at 10% A for 2.1 min. For LC condition, 5.00 μL of sample was injected under the column temperature of 50°C at 0.4 mL/min of elution flow rate. Drying gas of N_2 was supplied at the flow rate of 4 L/min along with drying gas temperature of 230°C; nebulizer pressure of 15 psi, capillary voltage of 2500 V for both positive and negative were set as MS conditions. Fragmentor voltage ranging from 60 to 200 V, collision energy range from 5 to 60 eV, and cell accelerator voltage of 4 V were set for data acquisition of individual PFAS.

For wastewater, river and tap water samples, solid phase extraction (SPE) was conducted by adding internal standard solution of known concentration (1 μg/L) in the purpose of accurate measurement while concentrating PFAS. SPE procedure was performed using Oasis WAX for PFAS analysis (150 mg, 6 cc, 30 μm) from Oasis ® (USA). 4 mL of methanol and 4 mL of water were used for conditioning process. The sample was then loaded at at flow rate of 5 mL/min. After loading step, cartridge was washed with 10 mL of water and 5 mL of 20% methanol in water, and dried completely under 15 mmHg vacuum. PFASs were eluted with 10 mL of 0.1% ammonium hydroxide in methanol. Finally, the eluted solution was concentrated to 0.5 mL by nitrogen evaporator at 60°C.

Cation (Na^+, Si, K^+, Ca^{2+}, Mg^{2+}) and anion (F^-, Cl^-, Br^-, NO_3^-,PO_4^{3-}, SO_4^{2-}) in wastewater were analyzed by inductively coupled plasma mass spectrometry (7800 ICP-MS, Agilent Technologies, USA), inductively coupled plasma optical emission spectroscopy (iCAP 7000 Series, Thermo Fisher Scientific, USA), and ion chromatography (Metrohm® 925 ECO

IC, Metrohm, Switzerland), respectively. The total organic carbon (TOC) content of a wastewater sample was measured using a TOC analyzer (ASI-V, Shimadzu, Japan).

Text S7. Performance of Aptamer-based qPCR Method in Tap Water and River Water.

To further investigate the potential interference effects more systematically, the recovery test was carried out using two more additional real water samples: tap water and river water collected from Geumgang River, South Korea. LC-MS/MS analysis revealed background PFOS levels of 2.3 ± 0.047 ng/L and 7.2 ± 5.5 ng/L, respectively, along with the presence of other PFASs, especially those with a total of C–F chain ≥ 6 (17.5 and 21.8 ng/L; Table S7). Following PFOS spiking at 30, 60, and 90 ng/L to present higher level of PFOS among the others, aptamer-based qPCR method yielded recovery rates with less than 12.5% deviation (Table S8).

In addition, the river water sample exhibited TOC levels of 4.6 ± 0.16 mg/L as C, which is more than 2-fold that of the industrial wastewater sample. However, no significant changes in recovery were observed, implying negligible influence from organic matter. To assess the impact of TDS, NaCl concentrations were varied (0, 500, and 4,000 mg/L) in deionized water (DI) to reflect the TDS range of the industrial wastewater, at a fixed PFOS concentration of 30 pM (Figure S19). The resulting qPCR signal changes (Ct/Ct_0) showed only ~8% maximum deviation across the salt conditions indicating minimal impact on detection accuracy under the current buffer system. Ultimately, these results further support that the deviation observed in industrial wastewater may be primarily attributable to coexisting long-chain PFASs (C–F chain ≥ 6). The qPCR-based aptasensor exhibits robust measurement at the picomolar level even under these complex and challenging environmental conditions.

Table S1. The information of sequences used in SELEX procedure.

Type	Length	Sequence (5' → 3')	Modification
Random Sequence (ssDNA)	72 nt	GGAGGCTCTCGGGACGAC(N_{30})GTCGTCCCGATGCTGCAATCGTAA	None
Reverse Primer	22 nt	/5-Biotin/TTACGATTGCAGCATCGGGACG	Biotin
Forward Primer	18 nt	GGAGGCTCTCGGGACGAC	None
Capture DNA (cDNA)	18 nt	GTCGTCCCGAGAGCCATA/3BioTEG/	Biotin

Table S2. The concentration used during the SELEX along with subsequent rounds with comparison of previous condition.

Round	PFOS (this study)
1	100 μM PFOS
2	50 μM PFOS
3	50 μM PFOS
4	10 μM Counter, 50 μM PFOS
5	10 μM Counter, 50 μM PFOS
6	10 μM Counter, 50 μM PFOS
7	20 μM Counter, 50 μM PFOS
8	20 μM Counter, 50 μM PFOS
9	20 μM Counter, 50 μM PFOS

Table S3. Most abundant 7 sequences discovered via SELEX with dissociation constants. Uncertainties represent standard deviation of experimental triplicates.

Candidate	Sequence (5' → 3')	Dissociation constant (K_D in μM)
PFOS_JYP_1	CTC TCG GGA CGA CGG GTA GTC TGT GTA TGC TCG CAA GCG TGC TGT CGT CCC	6.93 ± 0.15
PFOS_JYP_2	CTC TCG GGA CGA CGG GGT AAC GGT TTG ATC TCC TTC GTG TTC TGT CGT CCC	6.76 ± 0.20
PFOS_JYP_3	CTC TCG GGA CGA CGG GGG TTG CAT CTT TAT ATG TTT CTT CTC TGT CGT CCC	7.82 ± 0.19
PFOS_JYP_4	CTC TCG GGA CGA CGG GGG GCT ATT TCT TCA TTC TCT GCT GAC TGT CGT CCC	6.87 ± 0.06
PFOS_JYP_5	CTC TCG GGA CGA CGG CCA GAT GGA GCT TTT ATT TCC CTT CCT CGT CGT CCC	7.33 ± 0.21
PFOS_JYP_6	CTC TCG GGA CGA CGG CAG CTC AGC ATC TTT GTT GCC CCT GAC TGT CGT CCC	7.69 ± 0.04
PFOS_JYP_7	CTC TCG GGA CGA CCA CCA GGT CTC GTC TTA TTG GCT ATG TGT TGT CGT CCC	8.42 ± 0.34

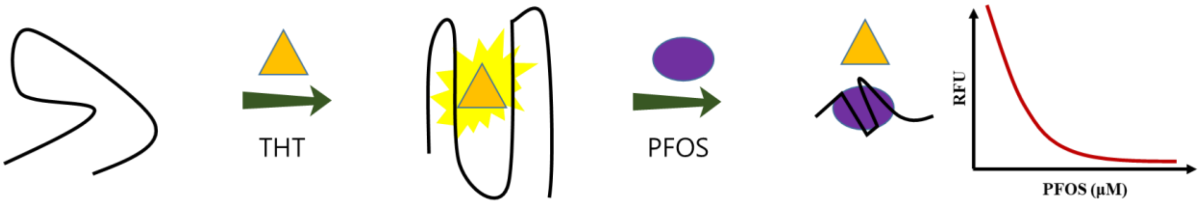

Scheme S1. Illustration of fluorescence based ThT aptasensor.

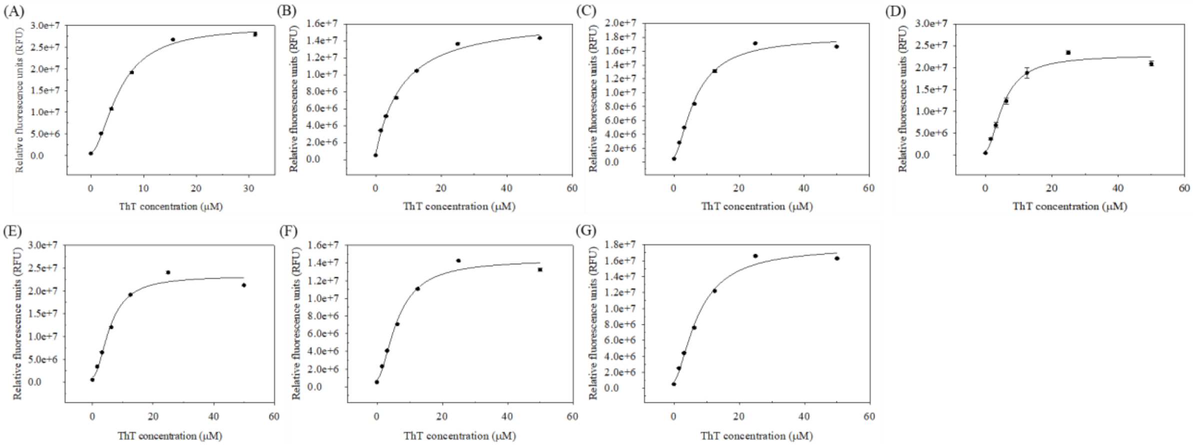

Figure S1. Fluorescence responses for ThT (0 – 50 µM) binding test with fixed concentration of 400 nM (A) PFOS_JYP_1, (B) PFOS_JYP_2, (C) PFOS_JYP_3, (D) PFOS_JYP_4, (E) PFOS_JYP_5, (F) PFOS_JYP_6, and (G) PFOS_JYP_7. The plots were fitted with Hill's equation. Error bars represent the standard deviation of experimental triplicates. The solution pH was held in 7.5.

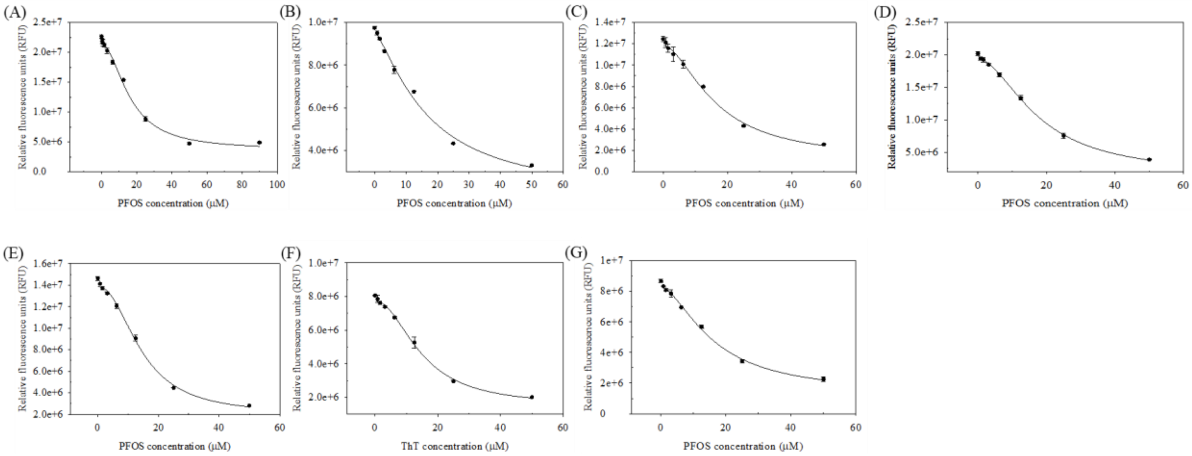

Figure S2. Fluorescence responses for PFOS (0 – 50 µM) binding test with fixed concentration of 400 nM (A) PFOS_JYP_1, (B) PFOS_JYP_2, (C) PFOS_JYP_3, (D) PFOS_JYP_4, (E) PFOS_JYP_5, (F) PFOS_JYP_6, and (G) PFOS_JYP_7. Concentration of 10 µM ThT was used for all the experiments. The plots were fitted with Hill's equation. Error bars represent the

standard deviation of experimental triplicates. The solution pH was held in 7.5.

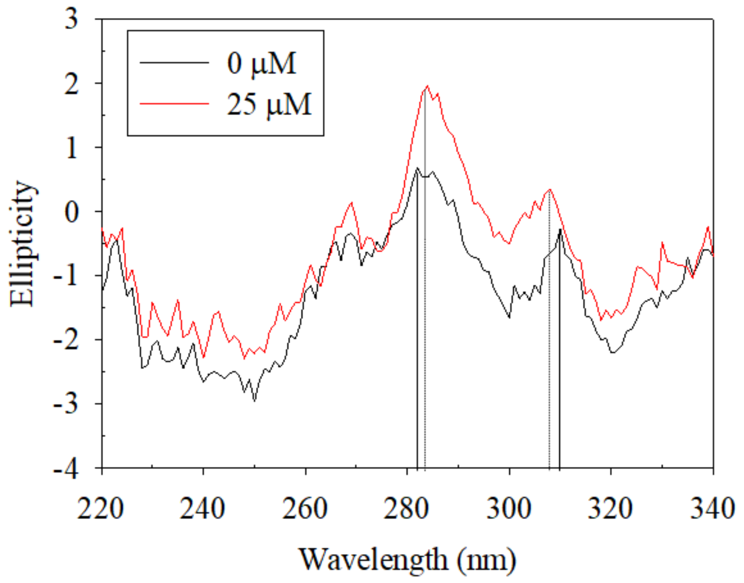

Figure S3. Circular dichroism spectroscopy of PFOS_JYP_2 with/without PFOS.

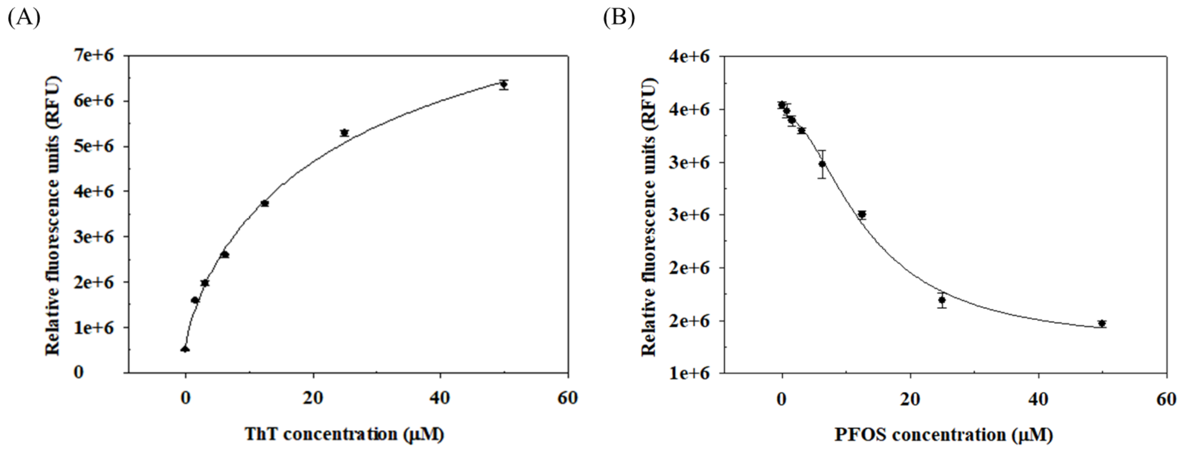

Figure S4. Fluorescence responses for (A) ThT (0 – 50 µM) binding test with fixed concentration of 400 nM small upper loop of PFOS_JYP_2, and (B) PFOS (0 – 50 µM) binding test with fixed concentration of 400 nM small upper loop of PFOS_JYP_2 PFOS with the concentration of 10 µM ThT. The plots were fitted with Hill's equation. Error bars represent the standard deviation of experimental triplicates. The solution pH was held in 7.5.

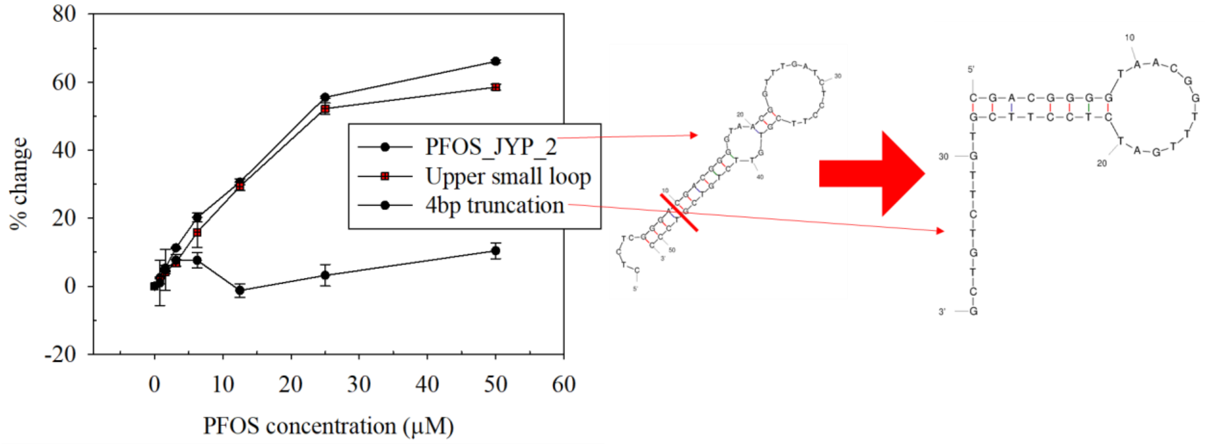

Figure S5. The changes in fluorescence responses were compared between PFOS_JYP_2 and sequence with shortened flanking sequence to yield 4 bp. The fluorescence response was measured at the pH of 7.5. 0 – 50 µM PFOS was incubated with 400 nM each aptamer and 10 µM ThT. Error bars represent standard deviation of experimental triplicates.

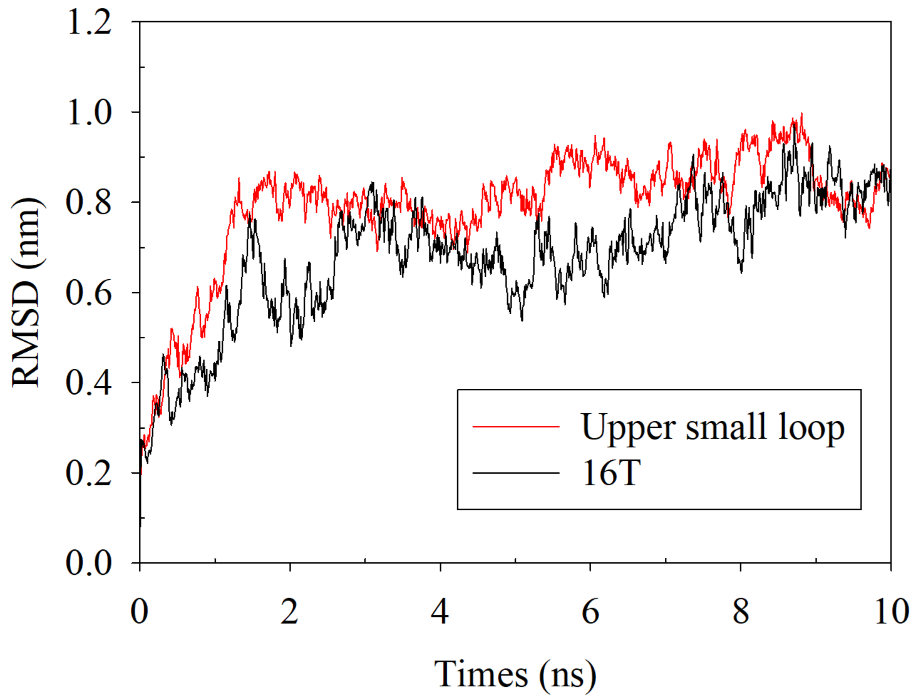

Figure S6. RMSD analysis during MDS of tertiary structure of aptamer binding site.

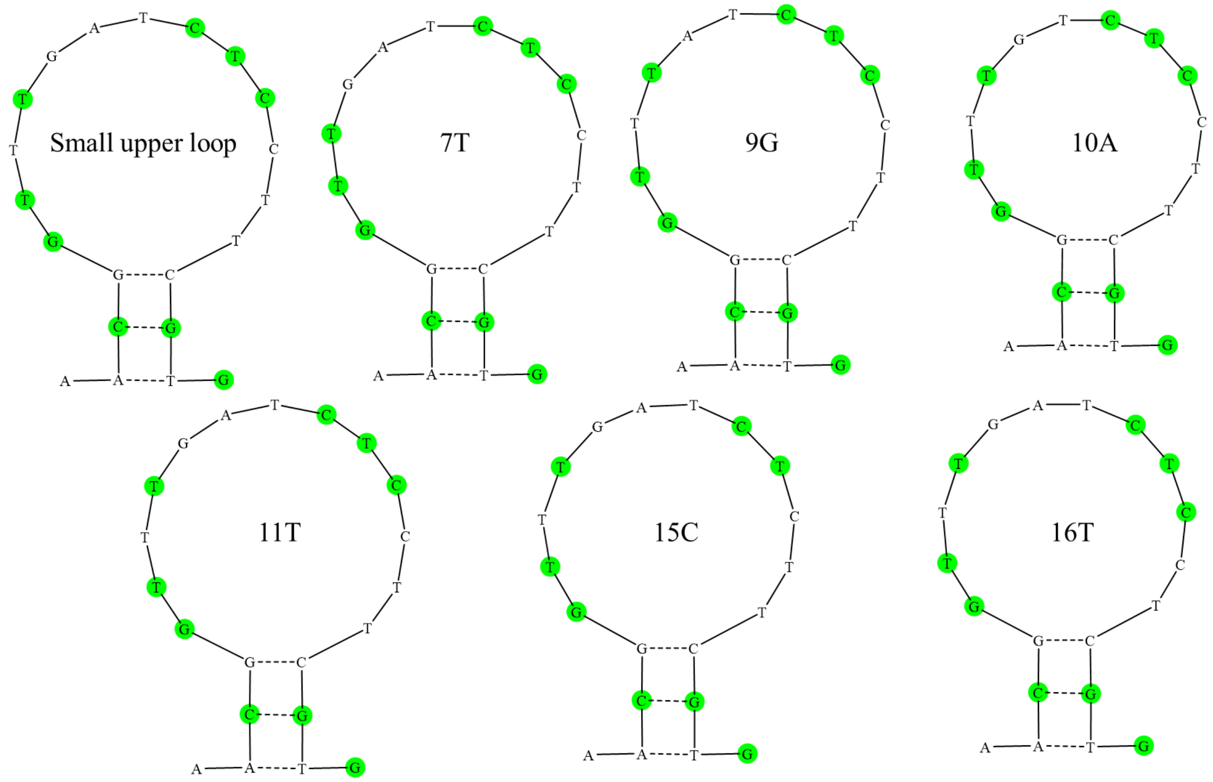

Figure S7. Predicted secondary structures for small upper loop, 7T, 9G, 10A, 11T, 15C, and

16T aptamers with binding sites high lightened with green shading. The secondary structures were predicted via MFold[7].

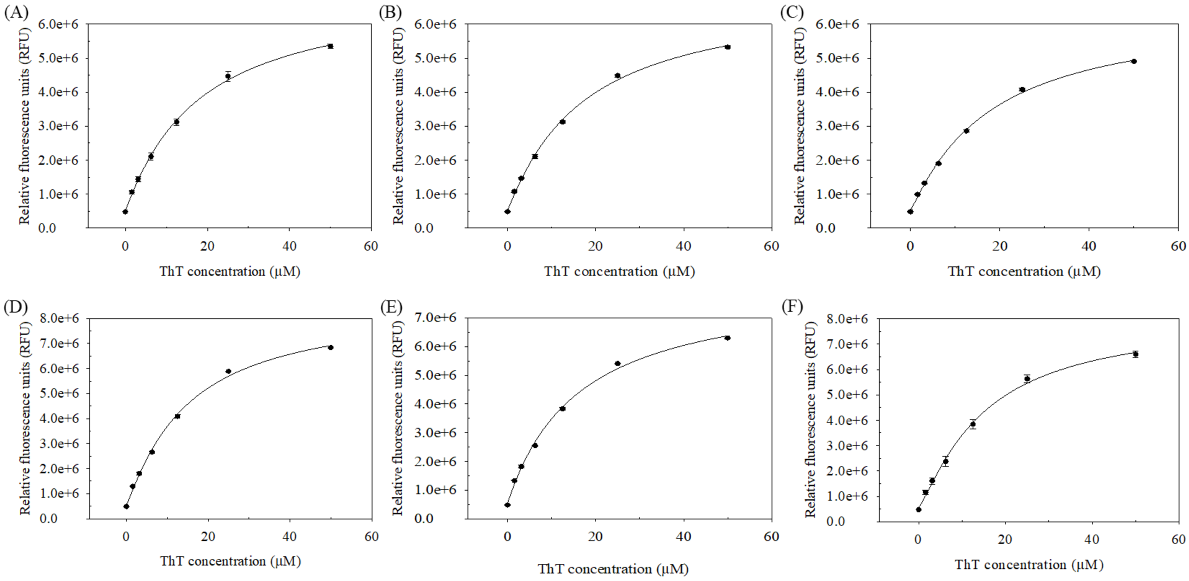

Figure S8. Fluorescence responses for ThT (0 – 50 µM) binding test with fixed concentration of 400 nM (A) 7T, (B) 9G, (C) 10A, (D) 11T, (E) 15C, and (G) 16T. The plots were fitted with Hill's equation. Error bars represent the standard deviation of experimental triplicates. The solution pH was held in 7.5.

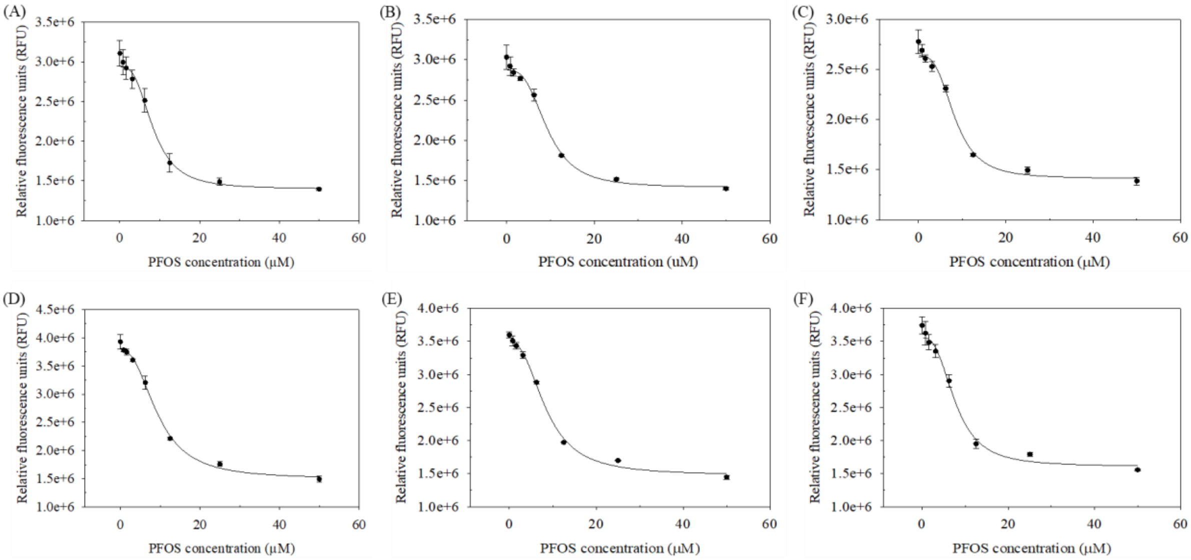

Figure S9. Fluorescence responses for PFOS (0 – 50 µM) binding test with fixed concentration of 400 nM (A) 7T, (B) 9G, (C) 10A, (D) 11T, (E) 15C, and (G) 16T. Concentration of 10 µM ThT was used for all the experiments. The plots were fitted with Hill's equation. Error bars represent the standard deviation of experimental triplicates. The solution pH was held in 7.5.

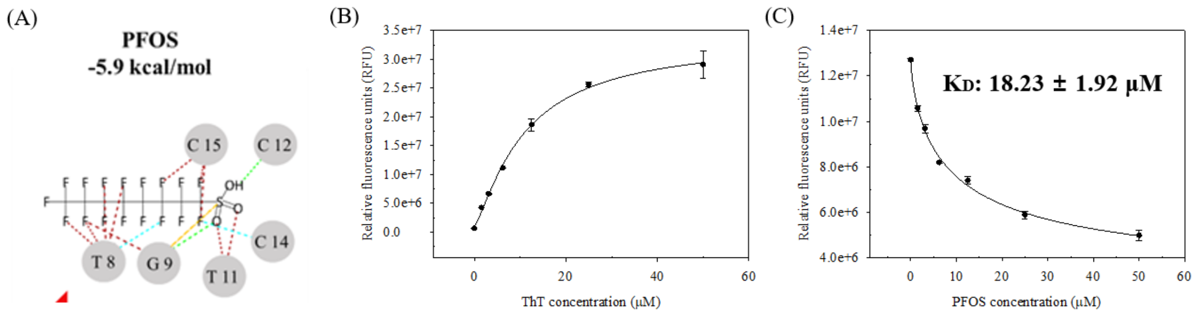

Figure S10. (A) Illustration of the aptamer bases directly interacting with PFOS and the specific binding positions involved in the interaction, Fluorescence responses for (B) ThT (0 – 50 µM) binding test with fixed concentration of 400 nM small upper loop of PFOS_JYP_2 variant in which the 9th guanine was mutated to cytosine. and (C) PFOS (0 – 50 µM) binding test with fixed concentration of 400 nM small upper loop of PFOS_JYP_2 PFOS with the concentration of 10 µM ThT. The plots were fitted with Hill's equation. Error bars represent the standard deviation of experimental triplicates. The solution pH was held in 7.5.

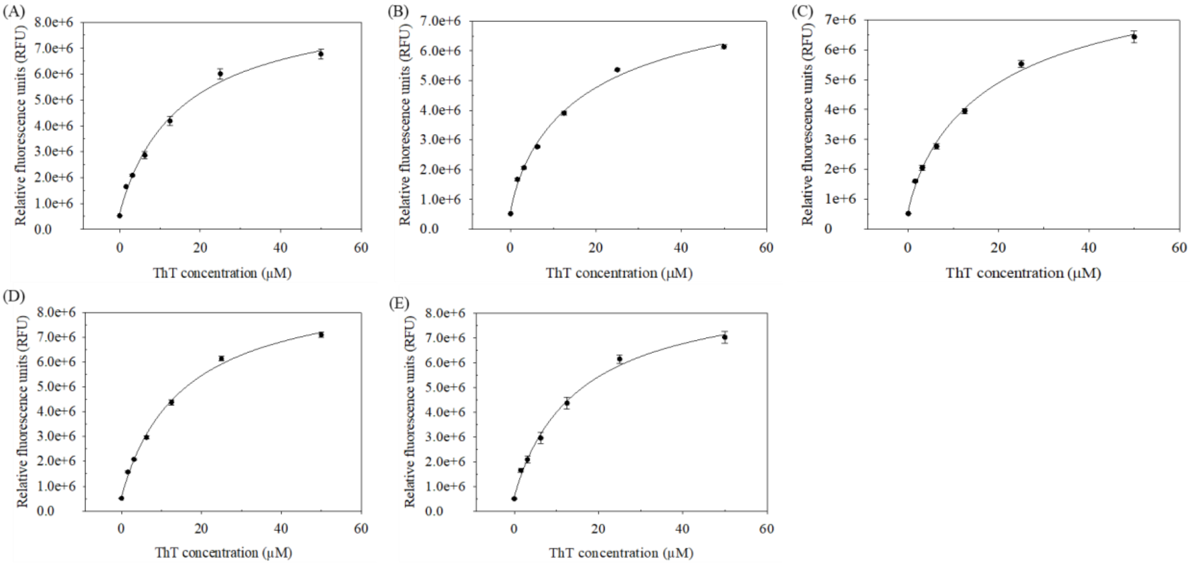

Figure S11. Fluorescence responses for ThT (0 – 50 µM) binding test with fixed concentration of 400 nM (A) 7T, 16T, (B) 9G, 16T, (C) 10A, 16T, (D) 11T, 16T, and (E) 15C, 16T. The plots were fitted with Hill's equation. Error bars represent the standard deviation of experimental triplicates. The solution pH was held in 7.5.

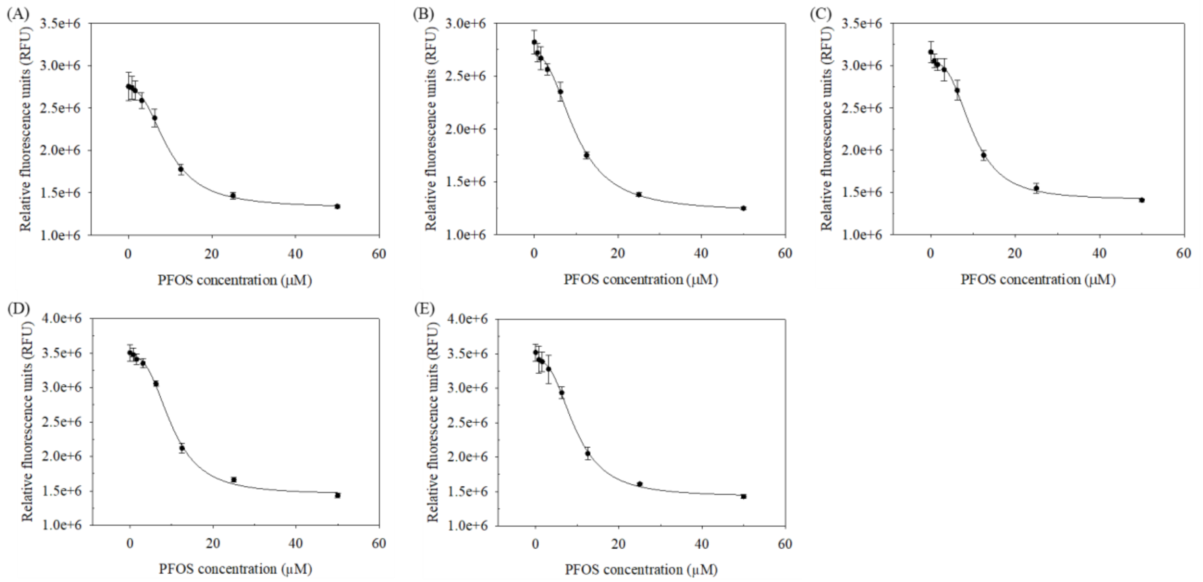

Figure S12. Fluorescence responses for PFOS (0 – 50 μM) binding test with fixed concentration of 400 nM (A) 7T, 16T, (B) 9G, 16T, (C) 10A, 16T, (D) 11T, 16T, and (E) 15C, 16T. Concentration of 10 μM ThT was used for all the experiments. The plots were fitted with Hill's equation. Error bars represent the standard deviation of experimental triplicates. The solution pH was held in 7.5.

Table S4. Values of dissociation constant (K_D) for the truncated aptamers. Uncertainties represent standard deviation of experimental triplicates.

**PFOS_JYP_2**	**Dissociation constant (K_D in μM)**
7T	7.99 ± 0.50
9G	8.86 ± 0.53
10A	8.30 ± 0.65
11T	8.60 ± 0.27
15C	7.57 ± 0.12
16T	6.77 ± 0.16
Upper small loop	11.21 ± 0.16
Original	6.76 ± 0.20

Table S5. Values of dissociation constant (K_D) for the listed sequences. Uncertainties represent standard deviation of experimental triplicates.

PFOS_JYP_2	Dissociation constant (K_D in μM)
7T, 16T	7.97 ±0.49
9G, 16T	7.35 ± 0.42
10A, 16T	8.19 ± 0.04
11T, 16T	8.82 ± 0.37
15C, 16T	7.86 ± 0.18
16T	6.77 ± 0.16
Upper small loop	11.21± 0.16
Original	6.76 ± 0.20

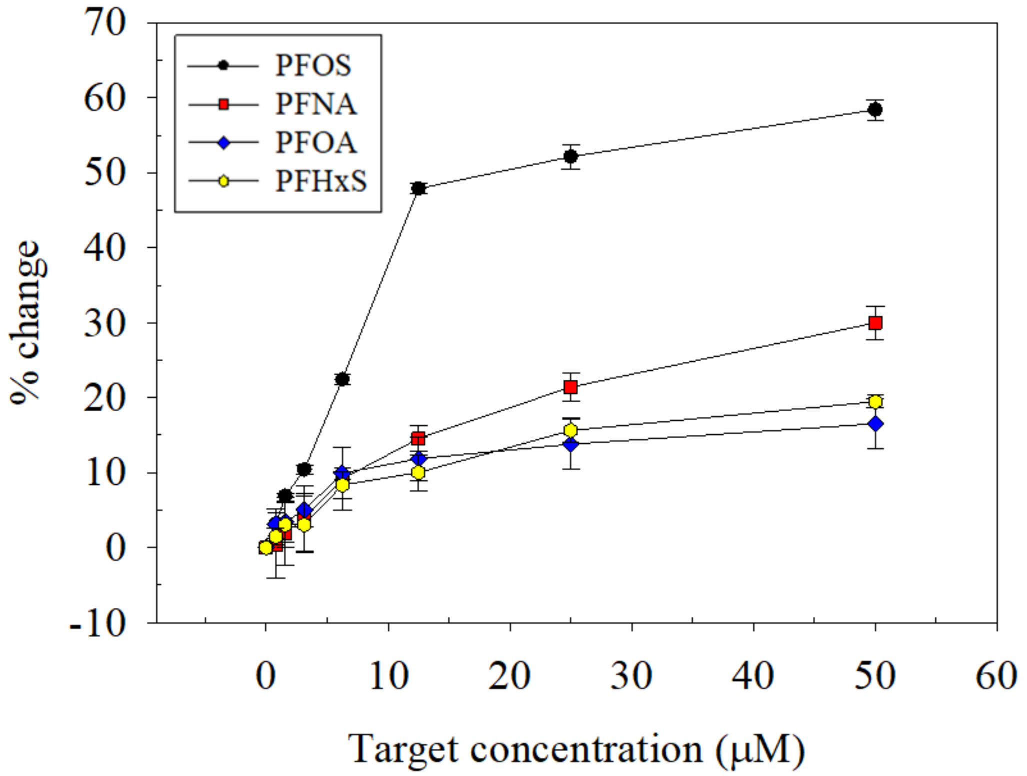

Figure S13. Changes in fluorescence responses over the range of 0 – 50 μM PFOS, PFNA, PFOA, and PFHxS at the fixed 16T aptamers concentration of 400 nM and 10 μM ThT. The solution was maintained at the pH of 7.5 with error bars from the experimental triplicates.

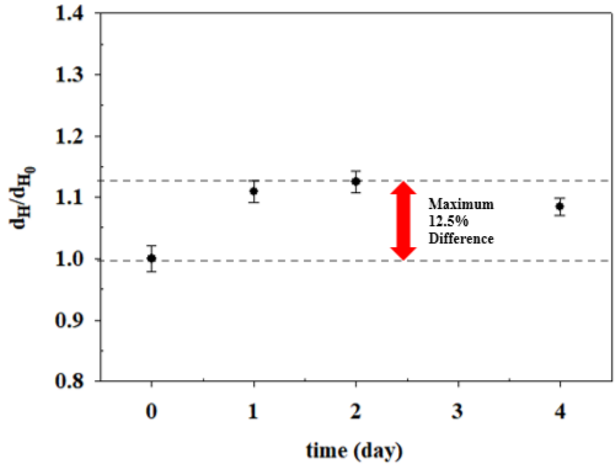

Figure S14. Changes in size of aptamer–GNP complexes (100 pmole of aptamer) compared to day 0 (first day of incubation) over 5 days. Uncertainties represent the standard deviation of experimental triplicates.

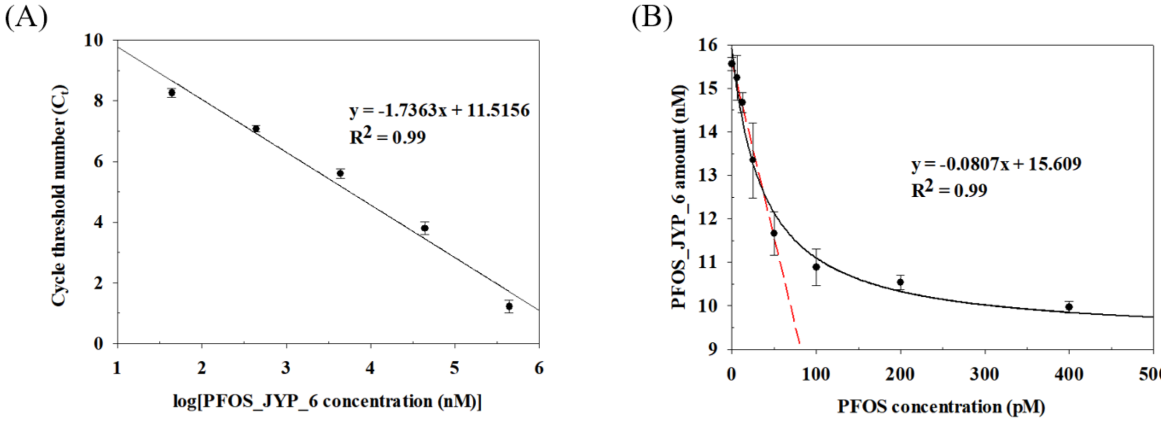

Figure S15. (A) qPCR standard curve generated from 1:2 serial dilutions of known aptamer concentrations (3.125–50 nM), showing a strong linear relationship between log-transformed PFOS_JYP_6 aptamer concentrations and Ct values. (B) Correlation curve demonstrating the inverse relationship between PFOS concentration (0–400 nM with 1:2 serial dilution) and unbound aptamer concentration in the supernatant, validating the feasibility of indirectly deducing PFOS concentration via qPCR-based aptamer quantification.

Table S6. Measurement of average size of the GNP after addition of 100 pmole of aptamer and 200pM of PFOS using DLS. Uncertainties represent standard deviation of experimental triplicates.

Aptamer		Average d_H (d.nm)
**PFOS_JYP_6**	Aptamer–GNP complexes	299.4 ± 39.1
	After PFOS incubation	590.6 ± 44.6
**PFOS_JYP_2**	Aptamer–GNP complexes	177.2 ± 10.9
	After PFOS incubation	193.6 ± 3.4

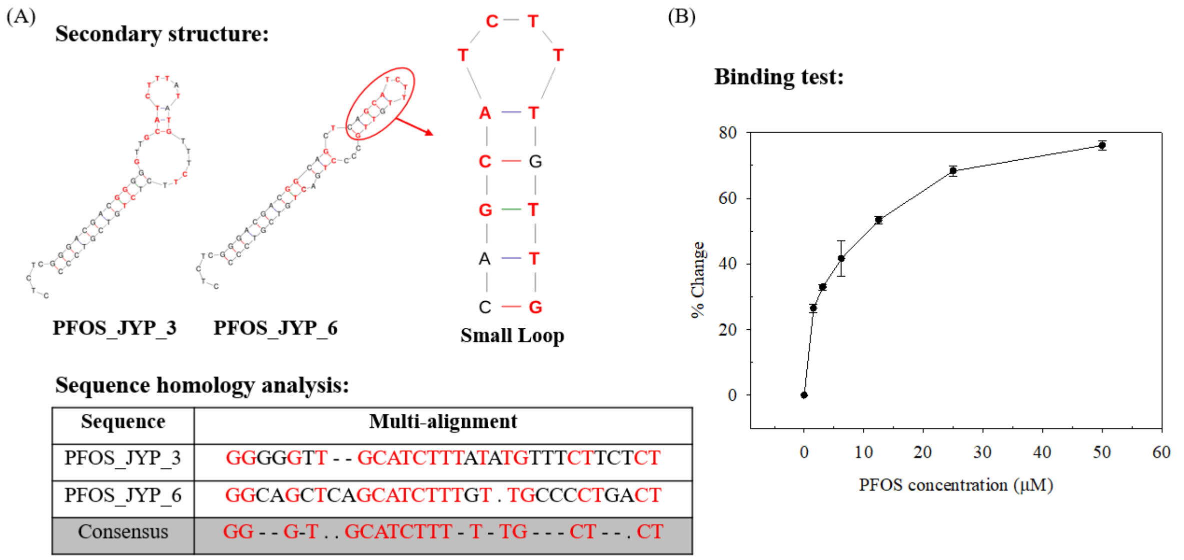

Figure S16. (A) Secondary structures predicted using the MFold web-based program[7]. Phylogenetic tree and sequence homology analyses between PFOS_JYP_3 and 6 (consensus sequences are marked in red) were performed using MultAlin[11]. (B) Changes in the fluorescence responses of the small loop of PFOS_JYP_6 at a pH of 7.5. 0–50 μM PFOS was incubated with each aptamer (400 nM) and 10 μM ThT. Error bars represent the standard deviation of experimental triplicates.

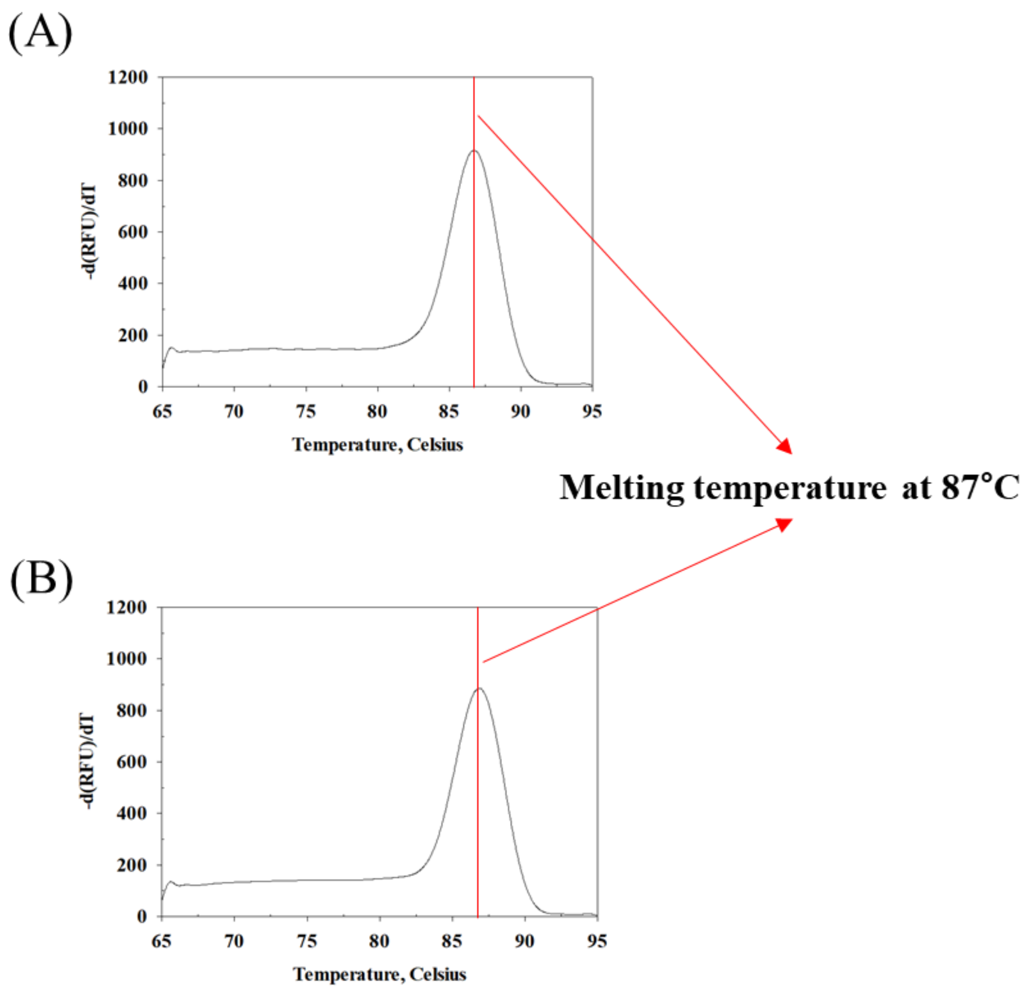

Figure S17. Melting curve of qPCR amplification of PFOS_JYP_6 (A) in the absence and (B) in the presence of GNPs using SYBR mode over the temperature ranging from 65°C to 95°C in the presence and absence of GNPs.

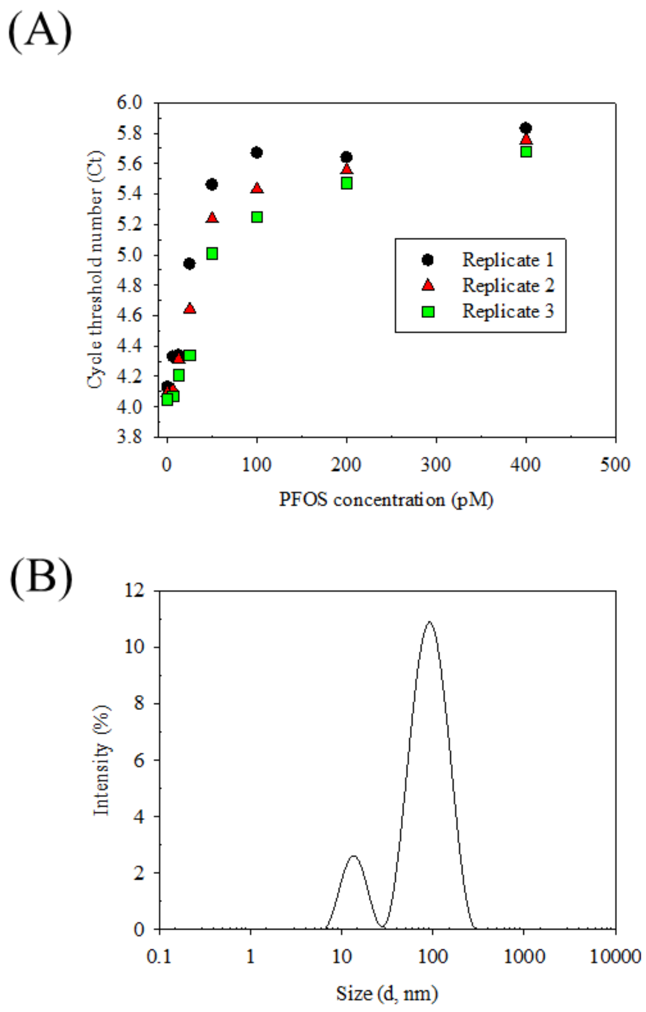

Figure S18. (A) The changes in the Ct responses in the PFOS concentration range of 0–400 pM for 100 pmol PFOS_JYP_6 for each replicate. (B) Dynamic light scattering (DLS) result of size distribution of synthesized GNPs used in qPCR measurement.

Table S7. The water matrix of the tap and river water (Geumgang River). PFAS, was analyzed using LC-MS/MS where the specific methods were provided in text S5. Uncertainties represent the standard deviation of experimental triplicates. Total dissolved solids (TDS) were calculated based on the conductivity multiplied by 0.64.

Water Sample	Category	Compounds	Values
**Tap water**	**PFAS**	PFBA (C–F of 3)	69 ± 3.1 ng/L
		PFBS (C–F of 4)	4.7 ± 0.78 ng/L
		PFHxA (C–F of 5)	1.6 ± 0.38 ng/L
		PFHpA (C–F of 6)	6.5 ± 0.87 ng/L
		PFHxS (C–F of 6)	2.7 ± 1.7 ng/L
		PFOA (C–F of 7)	4.5 ± 2.6 ng/L
		PFOS (C–F of 8)	2.3 ± 0.047 ng/L
		PFNA (C–F of 8)	1.7 ± 1.5 ng/L
		PFDA (C–F of 9)	2.1 ± 1.2 ng/L
	**TOC**		1.2 ± 0.084 mg/L as C
	**pH**		7.3
	**Conductivity (TDS)**		240 μS/cm, (150 mg/L)
**River water**	**PFAS**	PFBA (C–F of 3)	36 ± 13 ng/L
		PFBS (C–F of 4)	7.3 ± 4.3 ng/L
		PFHxA (C–F of 5)	2.6 ± 0.13 ng/L
		PFHpA (C–F of 6)	6.8 ± 0.18 ng/L
		PFHxS (C–F of 6)	1.1 ± 0.19 ng/L
		PFOA (C–F of 7)	3.2 ± 0.34 ng/L
		PFOS (C–F of 8)	7.2 ± 5.5 ng/L
		PFNA (C–F of 8)	1.8 ± 0.46 ng/L
		PFDA (C–F of 9)	1.7 ± 0.88 ng/L
	**TOC**		4.6 ± 0.16 mg/L as C
	**pH**		7.9
	**Conductivity (TDS)**		570 μS/cm (370 mg/L)

Table S8. Measurement of concentration after spiking PFOS in tap and river (Geumgang River) water via aptamer-based qPCR method. Uncertainties represent the standard deviation of experimental triplicates. The water composition regarding PFAS levels, TOC, TDS, and pH were shown in Table S7.

Water sample	Measured PFOS level via LC-MS/MS before spiking (ng/L)	Spiked PFOS level (ng/L)	Measured PFOS level via LC-MS/MS (ng/L)	Measured PFOS level via aptamer-based qPCR method (ng/L)	Recovery Rate ± RSD (%)
Tap water	2.3 ± 0.047	30	32.3	32.3 ± 0.90	100.0 ± 2.8
		60	62.3	69.9 ± 17.9	112.2 ± 25.6
		90	92.3	93.7 ± 6.5	101.5 ± 6.9
River water	7.2 ± 5.5	30	37.2	38.5 ± 1.0	104.0 ± 26.0
		60	67.2	63.4 ± 3.5	94.7 ± 5.5
		90	97.2	101.3 ± 7.8	112.5 ± 7.7

Table S9. Summary and comparison of aptamer-qPCR method with other reported PFOS sensors.

Method	Detection Range	LOD	Ref
Aptamer-qPCR method	2.9–50 ppt	2.9 ppt	This study
Fluorescence using complexation with porphyrin	0.025–8 ppm	4 ppb	[49]
Dye (HPTS) displacement with PFOS from chitosan	0.0025–1 ppm	500 ppt	[50]
Colorimetric using ethyl violet	10–1000 ppb	10 ppb	[51]
o-phenylenediamine (o-PD)-based MIP electrochemical sensor	4.75–750 ppb	20 ppt	[52]
Peroxisome proliferator-activated receptor α (PPARα)-based sensor	0.05–500 ppb	5 ppt	[53]

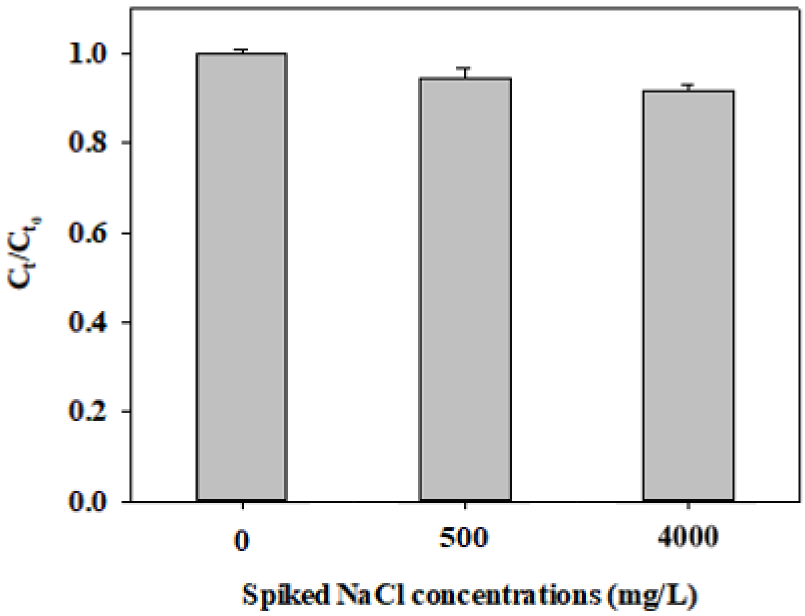

Figure S19. Effect of total dissolved solids (TDS) levels on qPCR performance. Different concentrations of NaCl (0, 500 and 4,000 mg/L) were used at the fixed PFOS concentration of 30 pM. The y-axis represents the ratio of the cycle threshold (Ct) value to that of the blank (Ct/Ct_0). Uncertainties represent the standard deviation of experimental triplicates.

REFERENCES

(1) Yang, K.-A.; Pei, R.; Stojanovic, M. N. In Vitro Selection and Amplification Protocols for Isolation of Aptameric Sensors for Small Molecules. *Methods* **2016**, *106*, 58–65. https://doi.org/10.1016/j.ymeth.2016.04.032.

(2) Park, J.; Yang, K.-A.; Choi, Y.; Choe, J. K. Novel ssDNA Aptamer-Based Fluorescence Sensor for Perfluorooctanoic Acid Detection in Water. *Environment International* **2022**, *158*, 107000. https://doi.org/10.1016/j.envint.2021.107000.

(3) Pramanik, S.; Nandy, A.; Chakraborty, S.; Pramanik, U.; Nandi, S.; Mukherjee, S. Preferential Binding of Thioflavin T to AT-Rich DNA: White Light Emission through Intramolecular Förster Resonance Energy Transfer. *J. Phys. Chem. Lett.* **2020**, *11* (7), 2436–2442. https://doi.org/10.1021/acs.jpclett.0c00237.

(4) Hanczyc, P.; Rajchel-Mieldzioć, P.; Feng, B.; Fita, P. Identification of Thioflavin T Binding Modes to DNA: A Structure-Specific Molecular Probe for Lasing Applications. *J. Phys. Chem. Lett.* **2021**, *12* (22), 5436–5442. https://doi.org/10.1021/acs.jpclett.1c01254.

(5) Hu, J.; Easley, C. J. A Simple and Rapid Approach for Measurement of Dissociation Constants of DNA Aptamers against Proteins and Small Molecules via Automated Microchip Electrophoresis. *Analyst* **2011**, *136* (17), 3461. https://doi.org/10.1039/c0an00842g.

(6) Trinh, K. H.; Kadam, U. S.; Rampogu, S.; Cho, Y.; Yang, K.-A.; Kang, C. H.; Lee, K.-W.; Lee, K. O.; Chung, W. S.; Hong, J. C. Development of Novel Fluorescence-Based and Label-Free Noncanonical G4-Quadruplex-like DNA Biosensor for Facile, Specific, and Ultrasensitive Detection of Fipronil. *Journal of Hazardous Materials* **2021**, 127939. https://doi.org/10.1016/j.jhazmat.2021.127939.

(7) Zuker, M. Mfold Web Server for Nucleic Acid Folding and Hybridization Prediction. *Nucleic Acids Research* **2003**, *31* (13), 3406–3415. https://doi.org/10.1093/nar/gkg595.

(8) Antczak, M.; Popenda, M.; Zok, T.; Sarzynska, J.; Ratajczak, T.; Tomczyk, K.; Adamiak, R. W.; Szachniuk, M. New Functionality of RNAComposer: Application to Shape the Axis of miR160 Precursor Structure. *Acta Biochim Pol* **2017**, *63* (4). https://doi.org/10.18388/abp.2016_1329.

(9) GROMACS Documentation. 632.

(10) Humphrey, W.; Dalke, A.; Schulten, K. VMD: Visual Molecular Dynamics. *Journal of Molecular Graphics* **1996**, *14* (1), 33–38. https://doi.org/10.1016/0263-7855(96)00018-5.

(11) Corpet, F. Multiple Sequence Alignment with Hierarchical Clustering. *Nucleic Acids Research* **1988**, *16* (22), 10881–10890. https://doi.org/10.1093/nar/16.22.10881.
